# Local Structure in α-BIMEVOXes (ME = Ge, Sn)

**DOI:** 10.1021/acs.chemmater.2c03001

**Published:** 2022-12-22

**Authors:** Yajun Yue, Aleksandra Dzięgielewska, Man Zhang, Stephen Hull, Franciszek Krok, Richard M. Whiteley, Harold Toms, Marcin Malys, Xuankai Huang, Marcin Krynski, Ping Miao, Haixue Yan, Isaac Abrahams

**Affiliations:** † Department of Chemistry, Queen Mary University of London, Mile End Road, LondonE1 4NS, United Kingdom; ‡ Faculty of Physics, Warsaw University of Technology, ul. Koszykowa 75, 00-662Warsaw, Poland; § School of Engineering and Materials Science, Queen Mary University of London, Mile End Road, LondonE1 4NS, United Kingdom; ∥ Science and Technology Facilities Council, ISIS Facility, Rutherford Appleton Laboratory, Chilton, Didcot, OxonOX11 OQX, United Kingdom; ⊥ Institute of High Energy Physics, Chinese Academy of Sciences, Beijing100049, China

## Abstract

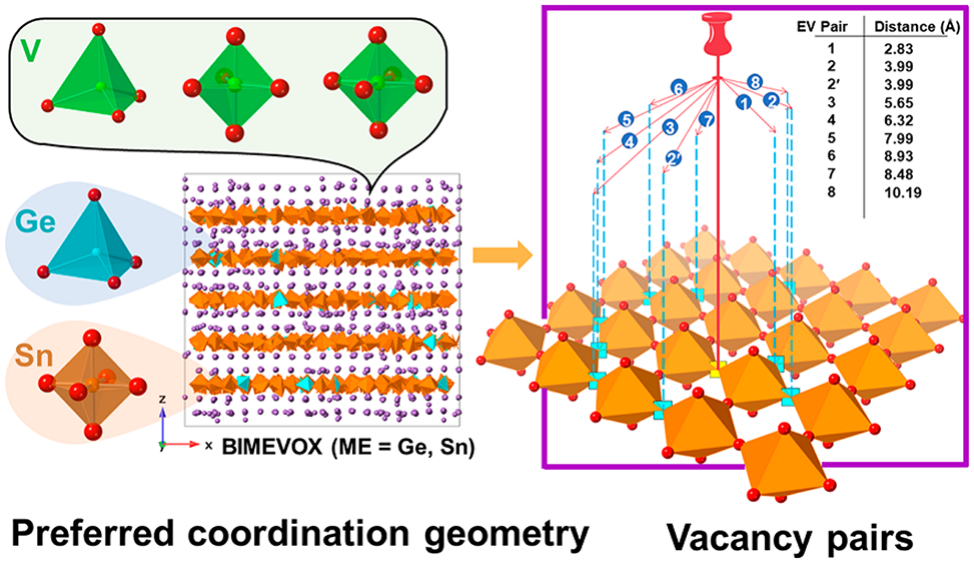

The BIMEVOXes are among the best oxide ion conductors at low and intermediate temperatures. Their high conductivity is associated with local defect structure. In this work, the local structures of two BIMEVOX compositions, Bi_2_V_0.9_Ge_0.1_O_5.45_ and Bi_2_V_0.95_Sn_0.05_O_5.475_, are examined using total neutron and X-ray scattering methods, with both compositions exhibiting the ordered α-phase at 25 °C and the disordered γ-phase at 700 °C. While the diffraction data for the α-phase do not allow for the polar (*C*2) and nonpolar (*C*2/*m*) structures to be readily distinguished, measurements of dielectric permittivity suggest the α-phase is weakly ferroelectric in character, consistent with calculations of spontaneous polarization based on a combination of density functional calculations and machine learning methodology. Reverse Monte Carlo (RMC) analysis of total scattering data reveals Ge preferentially adopts tetrahedral geometry at both temperatures, while Sn is found to predominantly adopt octahedral coordination in the α-phase and tetrahedral coordination in the γ-phase. In all cases, V polyhedra are found to consist of tetrahedral, pentacoordinate, and octahedral geometries, as also predicted by the crystallographic analysis and confirmed by ^51^V solid state NMR spectroscopy. Although similar long-range structures are observed at room temperature, the oxide ion vacancy distributions were found to be quite different between the two studied compositions, with a nonrandom deficiency in vacancy pairs in the second-nearest shell along the ⟨100⟩ tetragonal direction for BIGEVOX10, compared with a long-distance (>8.0 Å) ordering of equatorial vacancies for BISNVOX05. This is attributed to the differences in the preferred coordination geometries of the substituent cations in the two systems. Impedance spectroscopy measurements reveal both compositions show high conductivity in the order of 10^–1^ S cm^–1^ at 600 °C.

## 1 Introduction

Oxide ion conducting solids have important applications in oxygen sensors, oxygen separation devices and solid oxide fuel cells.^1−7^ The high ionic conductivity of the BIMEVOXes, particularly at intermediate temperatures (e.g., σ_600 °C_ ≈ 1.0 × 10^–1^ S cm^–1^ for the Cu substituted system,^8^) has led to a great deal of interest in these materials as electrolytes in such applications, with several studies of the structure–property relationships in the Bi_2_Me*^l^_*x*_*V_1–*x*_O_5.5–(5–*l*)*x*/2−δ_ (Me = dopants, *l* = valency) systems.^9−16^

**Figure 1:**
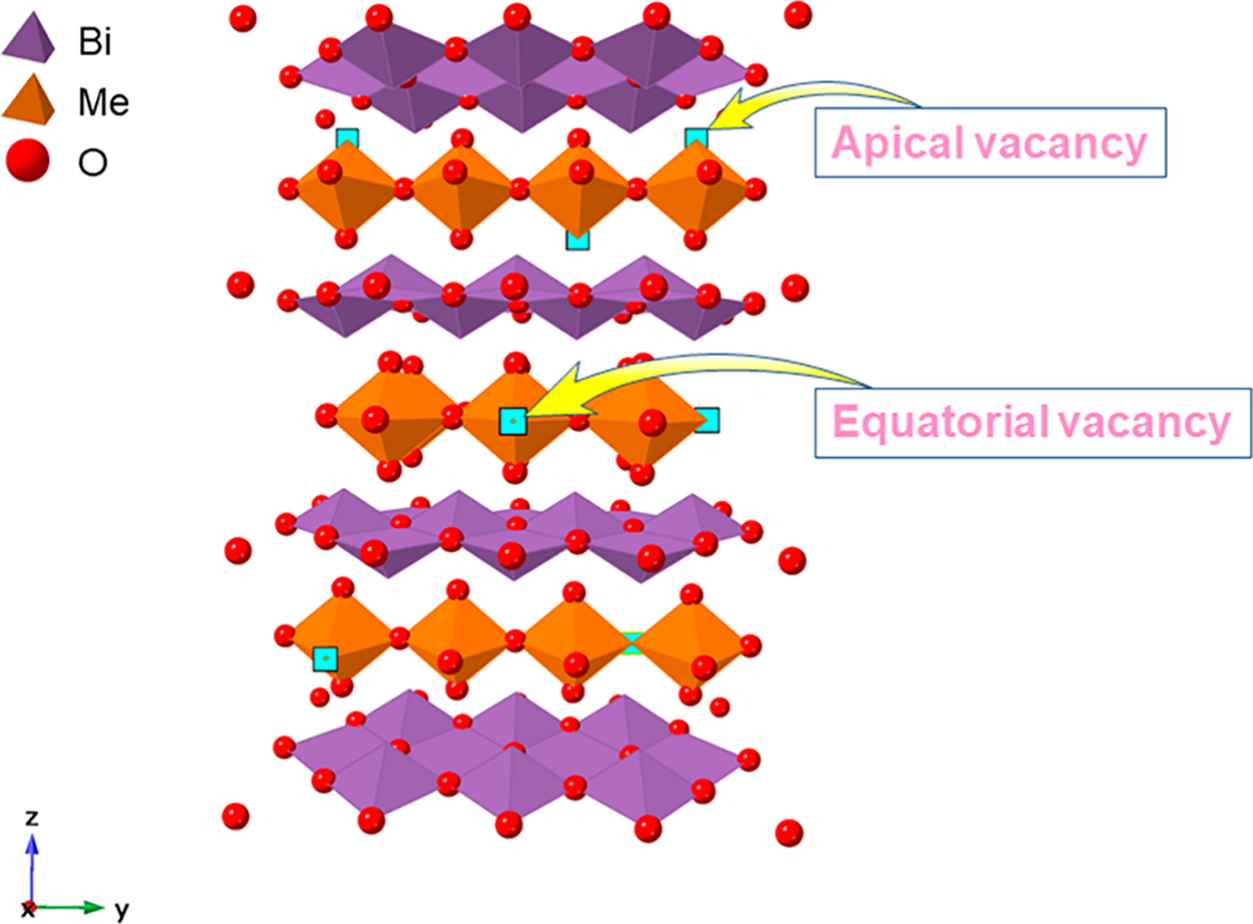
Idealized structure of γ-BIMEVOX showing the positions of apical and equatorial vacancies.

The crystal structures of the BIMEVOXes may be described as being derived from an ideal Aurivillius compound consisting of alternating bismuthate, (Bi_2_O_2_)_*n*_^2*n*+^, and metalate, (MO_4_)_*n*_^2*n*–^, layers (Figure 1). The parent compound, Bi_4_V_2_O_11−δ_,^17^ deviates from the ideal structure, in that the vanadate layer incorporates a large number of oxygen vacancies, i.e., (VO_3.5_*V*_0.5_)_*n*_^2*n*–^ (where *V* represents an oxide ion vacancy with respect to the ideal metalate layer). The degree of ordering of these oxygen vacancies varies with temperature and gives rise to the three main polymorphs, the monoclinic α-, orthorhombic β-, and tetragonal γ-phases. The lattice parameters of these polymorphs are often described as being related to an orthorhombic mean cell (*m*) of approximate dimensions *a*_m_ ≈ 5.53 Å, *b*_m_ ≈ 5.61 Å, and *c*_m_ ≈ 15.28 Å, such that *a*_*α*_ = 3*a*_m_, *b*_*α*_ = *b*_m_, *c*_*α*_ = *c*_m_; *a*_β_ = 2*a*_m_, *b*_β_ = *b*_m,_*c*_β_ = *c*_m_; and *a*_γ_ = *b*_γ_ ≈ *a*_m_/, *c*_γ_ ≈ *c*_m_.^12^ Substitution of V and/or Bi in Bi_4_V_2_O_11−δ_ leads to stabilization of the higher temperature polymorphs to room temperature, depending on the substituent and its concentration. The conductivity of BIMEVOX materials is closely associated with the level of disorder of the oxide ions in the vanadate layer, with the fully disordered γ-phase exhibiting the highest conductivity.^12^

Attention has therefore naturally focused on the γ-phase structures, with few studies of the more poorly conducting α- and β-phases. However, due to the similarities in their basic structure, studies of the lower symmetry ordered phases can reveal much about the nature of the local structure in the more interesting γ-phase, where details of the local structure are lost in the average structure due to the level of disorder. The structure of α-Bi_4_V_2_O_11−δ_ was first described in orthorhombic symmetry,^18,19^ but later studies revealed that the true symmetry was in fact monoclinic, with a β-angle very close to 90°.^20^ Careful electron diffraction studies revealed a 6*a*_m_ supercell; however, in the most accurate crystallographic study to date, the 3*a*_m_ subcell was used in space group *A*2,^13^ with some disorder in the vanadate layer remaining in the model. There are no similar studies on α-phase BIMEVOXes.

Analysis of local cation geometry, atomic disorder, vacancy distribution, and order–disorder behavior are of particular relevance to structural stability, to the structure–conductivity relationship, and in the prediction of new substitutional systems. Despite the importance of local structure in determining conductivity, there have been relatively few of these studies on Bi_4_V_2_O_11−δ_ and the BIMEVOXes. Detailed average structures derived from neutron diffraction have been used to suggest models of the defect structure,^16,21−24^ while more direct spectroscopic probes such as ^51^V solid-state nuclear magnetic resonance (NMR) and Raman spectroscopies as well as extended X-ray absorption fine structure (EXAFS) studies have yielded more direct evidence of local structure around cations in the vanadate layer.^25−28^ Recent developments in the analysis of total scattering data using reverse Monte Carlo (RMC) modeling have allowed for a detailed characterization of local structure in other oxide ion conducting solids such as Bi_3_YO_6_ and Bi_4_YbO_7.5_.^29,30^ Uniquely, the resulting models can be analyzed for physical evidence of vacancy ordering. Using these methods, we have recently found evidence for a nonrandom vacancy deficiency in the (100)_*m*_ direction in γ-BIGEVOX, consistent with the observed superlattice ordering in the α- and β-phases.^31^

We have previously proposed two limiting models for the defect structure in BIMEVOXes: the equatorial vacancy (EV) model, where all oxygen vacancies are located in the bridging equatorial positions, and the apical vacancy (AV) model, where the oxide ion vacancies are located in nonbridging apical positions (Figure 1). Substitution of vanadium by cations that have a different preferred coordination number can lead to polyhedral transformation in the remaining vanadium polyhedra, as seen in the BIGAVOX system where transformation of octahedral vanadium to pentacoordinate and tetrahedral vanadium occurs as more gallium is introduced into the system.^32^ In the EV model, the solid solution limit occurs when all the vanadium atoms have tetrahedral geometry or are completely substituted. The model successfully predicts solid solution limits for a number of BIMEVOX systems.^12^ In γ-BIMEVOXes, generally lower conductivity is observed with increasing substituent level and has been attributed to vacancy trapping effects.^16,33^ However, at lower levels of substitution, vacancy ordering leads to the stabilization of the α- and β-phases which exhibit lower levels of conductivity than the γ-phase. Nevertheless, these low substituent BIMEVOXes exhibit phase transitions to the highly conducting γ-phase at temperatures around 500 °C.

In the present work, we examine local structure and vacancy ordering in two tetravalent substituent BIMEVOX compositions, Bi_2_V_0.9_Ge_0.1_O_5.45_ and Bi_2_V_0.95_Sn_0.05_O_5.475_, using RMC analysis of total X-ray and neutron scattering data, supported by ^51^V and ^119^Sn solid-state NMR spectroscopy with conductivity examined using A.C. impedance spectroscopy. Both compositions exhibit the α-phase at room temperature and reversible α ↔ β and β ↔ γ phase transitions at high temperatures. Sn^4+^ is known to preferentially adopt octahedral geometry in oxide systems, while Ge^4+^ is typically tetrahedral. These differences in preferred coordination geometry result in differences in the vacancy ordering in these two crystallographically similar systems.

## 2 Experimental Section

### 2.1 Sample Preparation

Bi_2_V_0.90_Ge_0.10_O_5.45_ (BIGEVOX10) and Bi_2_V_0.95_Sn_0.05_O_5.475_ (BISNVOX05) were synthesized using a conventional solid-state method by grinding stoichiometric amounts of Bi_2_O_3_ (99.9%, Aldrich), V_2_O_5_ (98.0%, Avocado), and GeO_2_ (99.9%, Koch) or SnO_2_ (99.8%, Harrington Bros. Ltd.) powders (previously dried at 80 °C for 24 h) in an agate mortar with methylated spirits as a dispersant. The slurry was then dried at 80 °C in an oven, transferred to a platinum boat, and heated at 650 °C for 12 h. The powders were then quenched in air, reground, reheated to 850 °C for 24 h, and finally slow cooled to room temperature.

### 2.2 Characterization Methods

X-ray powder diffraction (XRD) was performed on a PANalytical X’Pert Pro diffractometer using Ni-filtered Cu Kα radiation (λ = 1.5418 Å) with an X’Celerator detector over a scan angle range from 5° to 120° in 2θ. The step width was set as 0.03342° with an effective collection time of 200 s per step. High temperature XRD experiments were performed on the same instrument with an Anton Paar HTK-16 furnace over the temperature range from 100 to 750 °C with a 50 °C interval on heating and cooling. A dwell time of 90 min at each temperature was controlled and repeat experiments showed no significant differences in transition temperatures.

For X-ray total scattering, all samples were sealed in quartz glass capillary tubes with an inner diameter of 1.5 mm, and data collected at the Diamond Light Source UK on the XPDF I15-1 beamline, using a synchrotron X-ray beam with a wavelength of 0.161669 Å and Na as the K_β_ filter at both 25 and 700 °C, with *ca*. 20 min between measurements to achieve thermal equilibrium.

Neutron powder diffraction was performed on the Polaris time-of-flight powder diffractometer at the ISIS Facility, Rutherford Appleton Laboratory. Data were collected over five detector banks, *viz*., backscattering (average angle 146.72°), 90° (average angle 92.59°), intermediate-angle (average angle 52.21°), low-angle (average angle 25.99°), and very low angle (average angle 10.40°) detectors, with the corresponding *d*-spacing ranges, 0.04–2.6 Å, 0.05–4.1 Å, 0.73–7.0 Å, 0.13–13.8 Å, and 0.3–48 Å, respectively. The powdered samples were initially sealed in an evacuated silica tube and then placed inside an 11 mm diameter vanadium can in an evacuated furnace. Data were collected at room temperature and from 300 to 700 °C in steps of 50 °C, with a dwell time of 10 min per step. At room temperature and 700 °C, data collections corresponding to proton beam charges of *ca*. 1000 μA h were made to allow for total scattering analysis, with shorter collections of 30 μA h acquired at all other temperatures. For total scattering data correction, diffraction data were collected on an empty silica tube inside an 11 mm diameter thin walled (0.05 mm wall thickness) vanadium can for *ca*. 200 μA h at room temperature and 700 °C.

Rietveld whole profile fitting was applied for structural refinements with the GSAS suite of programs,^34,35^ using both X-ray and neutron diffraction data. The α-phase structure was refined using three different models in space groups *Aba*2,^19^*C*2/*m*,^20^ and *C*2.^13^ The β-phase structure was refined using an orthorhombic model in space group *Amam*, with *a* = 11.2331 Å, *b* = 5.6491 Å, and *c* (stacking direction) = 15.3469 Å.^14^ The γ-phase structure was refined using a tetragonal model in space group *I*4/*mmm*, with *a* = 3.9274 Å and *c* (stacking direction) = 15.4274 Å.^22^ In the BIGEVOX10 sample, a small amount of BiVO_4_ (*ca*. 2.7 wt %) was observed and refined as a second phase using a monoclinic model (*I*2/*c*, *a* = 5.2196 Å, *b* = 11.7077 Å, *c* = 5.1079 Å, β = 90.633°).^36^ Details of the refinement process are given in the Supporting Information.

The reverse Monte Carlo (RMC) method using the RMCprofile software was applied to model local structure.^37,38^ The neutron total scattering structure functions, *S*(*Q*), and the total radial distribution functions, *G*(*r*), were produced using the software GudrunN, and the X-ray scattering function *F*(*Q*) was corrected using GudrunX.^39^ Fitting of the *S*(*Q*), *G*(*r*), and X-ray *F*(*Q*) functions was carried out with the measured neutron Bragg data used as a long-range order constraint. For the BIGEVOX10 sample, the initial model was constructed based on a 3*a*_m_ supercell of the ideal mean cell model, with *c* as the stacking direction. From this a 3 × 10 × 3 supercell was constructed for the RMC calculations. The chemical formula determined that a number of V atoms were randomly replaced by Ge. Similarly, the calculated number of oxygen vacancies was randomly or quasi-randomly introduced into equatorial positions in the vanadate layer. In the case of the quasi-random vacancy distribution, for BIGEVOX10 two vacancies were preferentially located around each Ge atom to ensure a coordination number of four was maintained for Ge in the starting model, while for BISNVOX05, vacancies were placed preferentially around vanadium atoms to maintain an initial octahedral geometry for Sn atoms. A soft bond valence summation (BVS) constraint^37^ was used along with a series of bond-stretching pseudopotential constraints for metal–oxygen pairs to avoid unrealistically short bonds. Cation swapping was tested but found to have no significant influence on the fits; therefore, only translational movements of atoms were permitted.

Simulation methods were used to estimate ionic charges in the structural models of BIGEVOX10 and BISNVOX05 to allow for the calculation of spontaneous polarization (*P*_s_). With configurations of *ca*. 10 000 atoms, the size of the structural models exceeds the limitations of typical *ab initio* methods. Therefore, a combination of density functional calculations, using the VASP package^40,41^ and machine learning methodology, was applied. For each composition ten initial configurations, based on the ideal structure, with different displacement of cations and oxygen vacancies were created, each containing two unit cells. Ten picoseconds of molecular dynamics simulations at 1500 K were performed within the canonical ensemble to sample the potential energy surface. For this, the exchange-correlation functional of Perdew–Burke–Ernzerhof (PBE)^42^ was used, along with a 400 eV cutoff energy for the plane-wave-basis set, a 10^–4^ global break condition for the electronic self-consistent loop, and 1 × 1 × 1 k-point sampling of the Brillouin zone. The resulting atomic positions were then used as starting points for the structural relaxation with similar settings. The optimized atomic positions were then used for single point simulations, using the PBE0 hybrid functional and 3 × 3 × 3 k-point sampling. The calculation of ionic charges was performed using the Bader partitioning scheme. The training and validation sets were constructed from the obtained charge values, as well as from the atomic environments of each atom, encoded using the Smooth Overlap of Atomic Positions descriptor.^43,44^ The machine learning model was created based on the Gaussian Process Regression approach, with the radial basis function kernel as implemented in Scikit-learn.^45^ Training and validation sets were weighted in a 0.8:0.2 ratio. During the validation of the obtained model, relatively low values of 0.012, 0.016, 0.019, 0.021, and 0.022 were obtained for the root-mean-square error of the elementary charges for Bi, Ge, Sn, V, and O ions, respectively. No significant outliers were observed. The obtained configurations of the RMC model containing predicted elementary charges were then folded back onto the crystallographic unit cell and then compared with the ideal centrosymmetric structural models to obtain the spontaneous polarization, *P*_s_, value along each lattice direction using equations of the type:
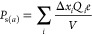
1summed over all atoms *i* in the unit cell, where *V* is the unit cell volume, Δ*x*_*i*_ is the distance parallel to the *a*-axis between the position of atom *i* in the folded relaxed configuration and the corresponding atom in the ideal centrosymmetric structure, *Q*_i_ is the partial ionic charge of atom *i* calculated using the Bader partitioning method, and *e* is the electronic charge. Similar equations were used to calculate values of *P*_s_ parallel to the *b*- and *c*-axes.

Magic angle spinning (MAS) ^51^V and ^119^Sn solid-state NMR data were collected at 157.85 and 223.79 MHz, respectively, on a Bruker AMX-600 spectrometer. For ^51^V measurements samples were loaded into a 2.5 mm diameter zirconia rotor and spun at 22 kHz. A pulse width of 1.0 μs was used, and 8192 points were acquired for each transient over 128 scans, with an acquisition time of 8.19 μs and a relaxation delay of 1 s. For ^119^Sn spectra, samples were loaded into a 4 mm diameter zirconia rotor and spun at 12 kHz. 8192 scans were acquired using the same pulse width and acquisition time with a relaxation delay of 30 s. ^51^V and ^119^Sn chemical shifts were referenced to external NH_4_VO_3_ (−575.7 ppm, 0.1 mol/L)^46^ and SnCl_4_ (−641.8 ppm, 0.3 mol/L)^47^ standard solutions, respectively. Isotropic resonances were modeled initially using DMfit,^48^ assuming a pseudo-Voigt peak shape. Whole spectral fitting was later carried out using the NMRLSS program.^49^

To measure electrical properties, the powder samples were pelletized at 150 MPa in a 13 mm diameter cylindrical die and then sintered at 800 °C for 8 h, followed by slow cooling. The obtained pellets were cut and then polished into blocks of approximate dimensions 2 mm × 3 mm × 5 mm and coated with platinum electrodes by cathodic discharge. Typically, impedance was measured on a Novocontrol Alpha analyzer with a ZG4 extension interface over the frequency range of 1.0 Hz to 1.0 MHz at temperatures from 100 °C up to 820 °C at intervals of 30 °C. Impedance was measured over two cycles of heating and cooling, with 1.0 h stabilization time at each temperature. The piezoelectric coefficient, *d*_33_, was measured using a Berlincourt *d*_33_ meter (ZJ-3B, China). Capacitance and loss tangent were measured using an impedance analyzer (Agilent, 4294A, Hyogo, Japan). High temperature dielectric permittivity and loss with respect to frequency were measured using an Agilent 4284A LCR meter over the temperature range 25–560 °C. Polarization versus electric field (*P*–*E*) and electrical current versus electric field (*I*–*E*) hysteresis loops were obtained with a ferroelectric hysteresis measurement tester (NPL, U.K.).

The composition of samples was confirmed by energy dispersive X-ray (EDX) analysis on synthesized powders using an FEI Inspect-F scanning electron microscope and confirmed the expected stoichiometry (Figure S1 and Table S1).

## 3 Results and Discussion

### 3.1 Phase Behavior

**Figure 2:**
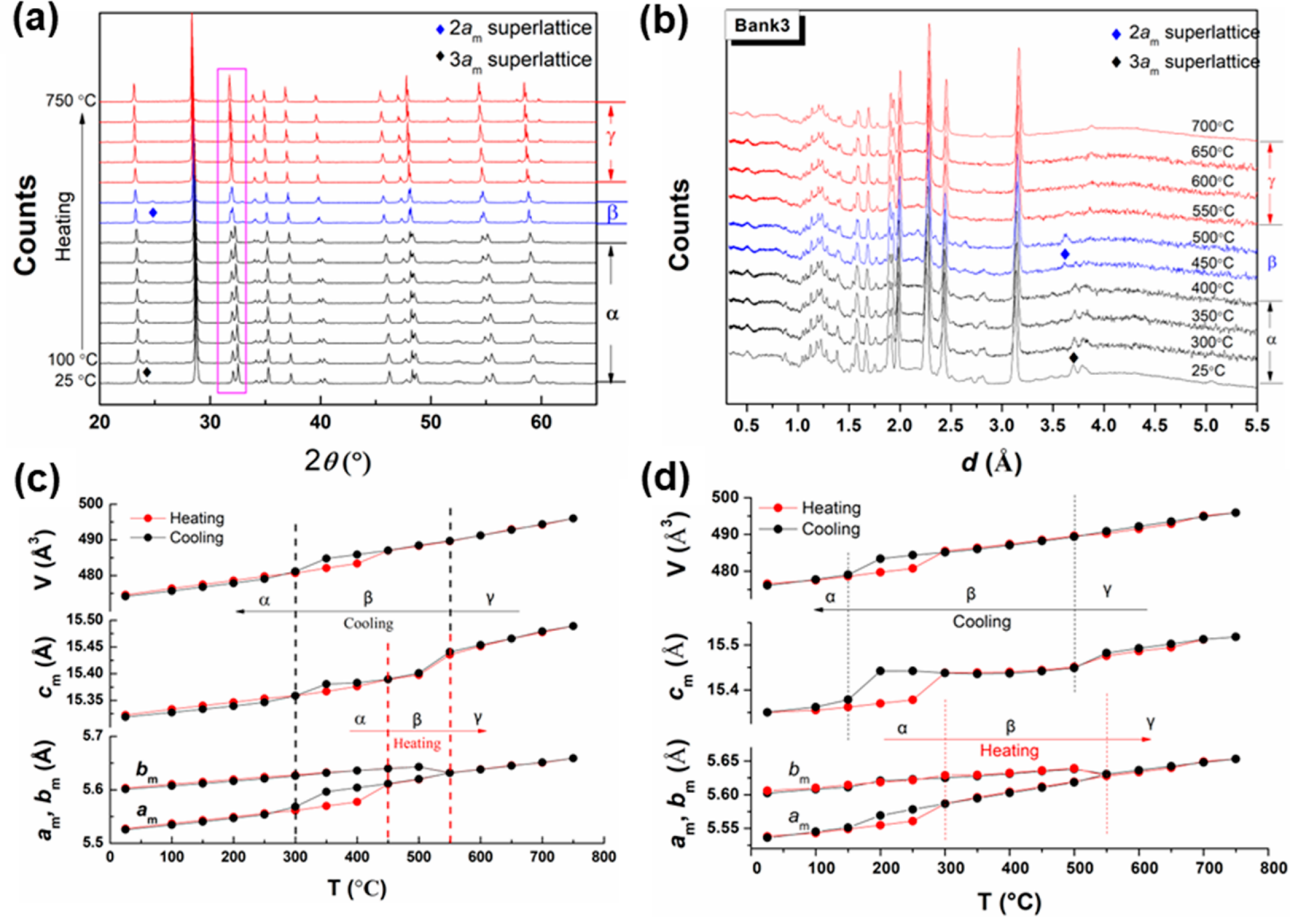
Thermal evolution of (a) X-ray and (b) neutron (bank 3) diffraction patterns for BIGEVOX10; (c and d) thermal variation of refined lattice parameters and mean cell volume for (c) BIGEVOX10 and (d) BISNVOX05 compositions.

Figure 2a shows the thermal variation of XRD patterns for BIGEVOX10 on heating from 25 to 750 °C, with the data on cooling shown in Figure S2a. Phase transitions are indicated by the variation of peaks around 32.2° 2θ (*d* ≈ 2.78 Å), corresponding to the (200) and (020) reflections in the mean cell model. On heating, the α → β transition is seen at *ca*. 450 °C, with the 3*a*_m_ superlattice peak (*ca*. 24°) shifting to the 2*a*_m_ reflection position (*ca*. 24.8°), corresponding to the shifting of the neutron diffraction peak at *d* ≈ 3.67 Å (Figure 2b, bank 3). At 550 °C, the structure transforms to a disordered γ-phase with the disappearance of the 2*a*_m_ superlattice peak. Meanwhile, the (200) and (020) reflections merge to form the tetragonal (110) peak (Figure 2a) as seen in the neutron diffraction patterns at *d* ≈ 2.78 Å (Figure S2b, bank 4). A similar thermal evolution of X-ray and neutron diffraction patterns (bank 4) for BISNVOX05 is shown in Figure S3, with clear α → β and β → γ phase transitions at 300 and 550 °C, respectively, the former being significantly lower than that for the corresponding transition in BIGEVOX10. It is noted here that the (220) reflection at *ca*. 46.1° 2θ (*d* ≈ 1.97 Å) is less split at room temperature in BISNVOX05 (Figure S3a) and BIGEVOX10 (Figure 2a) than in the parent compound Bi_4_V_2_O_11−δ_.^31^ This suggests that the room temperature structures of BISNVOX05 and BIGEVOX 10 show little or no monoclinic distortion compared to the unsubstituted parent compound.

Figure 2c,d shows the thermal variation of the equivalent mean cell lattice parameters and cell volume for BIGEVOX10 and BISNVOX05, respectively. Where possible, the neutron diffraction data were included in the Rietveld refinements. For both compositions, the *a*_m_ axis generally increases in length on heating, while a step corresponding to the α → β transition is observed for BIGEVOX10 between 400–450 °C and for BISNVOX05 between 250–300 °C. The *b*_m_-axis also increases linearly up to 500 °C, then decreases slightly, becoming equal to the *a*_m_-axis at the β → γ phase transition. Interestingly, in the β-phase region, the *c*_m_-axis shows subtle variations for BISNVOX05 on heating, in contrast to the increasing trend seen in BIGEVOX10. Thermal hysteresis is observed for both compositions in the *a*_m_-axis and the cell volume plots corresponding to the α ↔ β transition.

### 3.2 Conductivity Analysis

**Figure 3:**
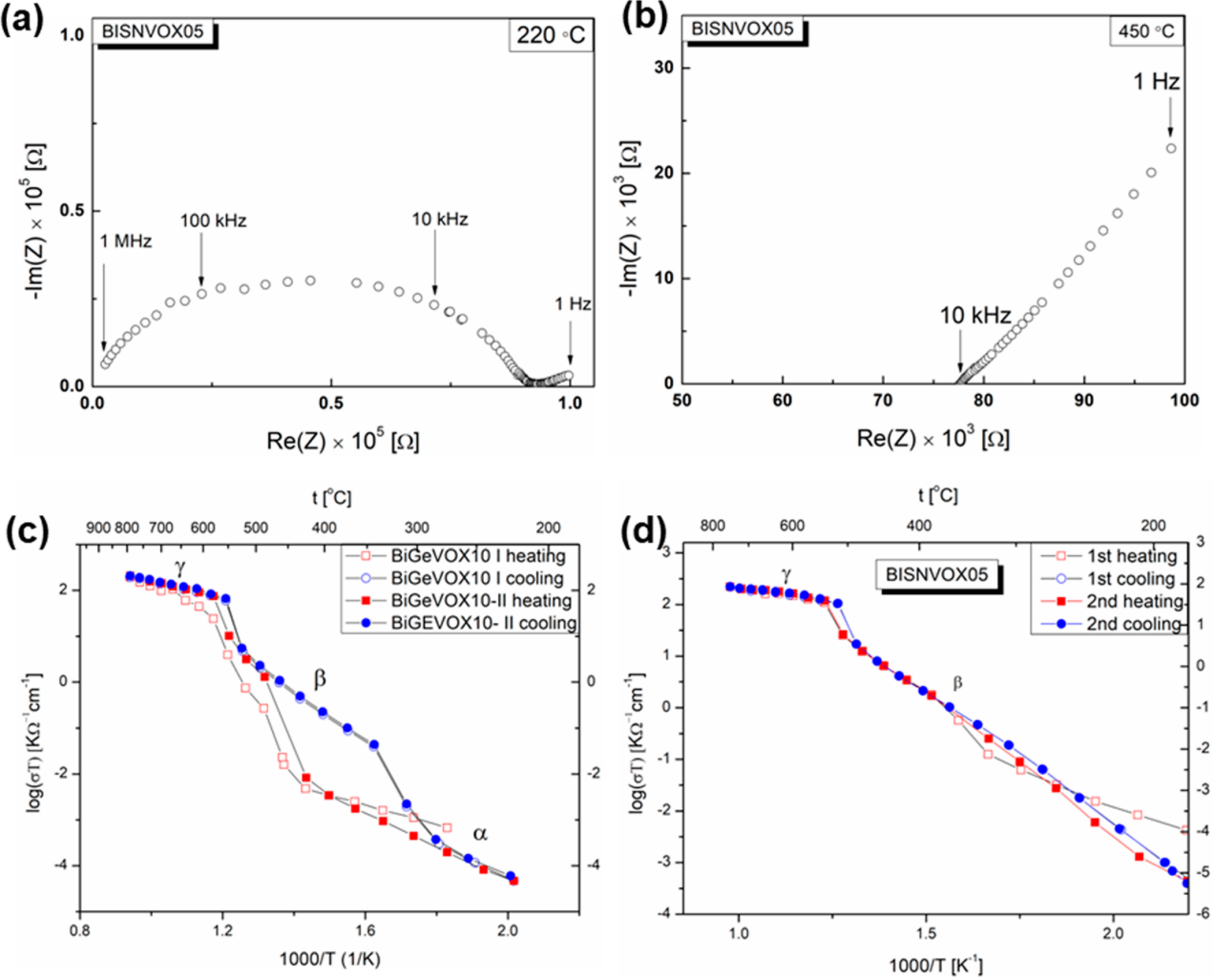
(a and b) Typical Nyquist plots at selected temperatures for BISNVOX05; Arrhenius plots of total conductivity for (c) BIGEVOX10 and (d) BISNVOX05 over two cycles of heating and cooling.

Figure 3a,b shows representative impedance spectra for the BISNVOX05 sample at 220 and 450 °C. At 220 °C, a depressed semicircle is observed at high frequencies, with a spur shown at lower frequencies. The intragrain and intergrain contributions are nonseparable, while the spur is associated with the interface between the electrolyte and the Pt electrode. At higher temperature (450 °C), the semicircle moves out of the frequency range, leaving only the spur. The observed spectra are similar to those of other BIMEVOXes, e.g., BICUVOX^8^ and BIMGVOX.^14^ Arrhenius plots of total conductivity for the BIGEVOX10 and BISNVOX05 compositions upon heating and cooling are shown in Figure 3c,d. The plot for BIGEVOX10 shows three linear regions in both heating and cooling processes. On heating, there is a jump in conductivity from around 430 °C, to a linear region of higher activation energy, followed by a second jump at around 550 °C to a linear region of low activation energy, respectively corresponding to the α → β and β → γ transitions, in agreement with the thermal phase evolution in Figure 2a. A large hysteresis is observed on cooling for BIGEVOX10, with the γ-phase region extending down to around 550 °C and the β-phase to around 350 °C. There are some differences between the first and second heating cycles. Of note is an apparent step in the first heating run between *ca*. 575 and 650 °C. This step is also seen in the parent compound, Bi_4_V_2_O_11_, and has been attributed to an intermediate phase, ε.^50^ Only on first heating are three linear regions seen in the Arrhenius plot for BISNVOX05, with subsequent heating and cooling runs showing only two linear regions corresponding to the β- and γ-phases. The α → β transition in the first cycle occurs at around 300 °C, in good agreement with the diffraction data (Figure 2b), but the large thermal hysteresis associated with this transition (as seen in BIGEVOX10, Figure 3c) and the fact that data were only collected to around 180 °C (below which data quality is generally poor) on cooling meant that the α-phase was not obtained after the first heating run.

Similar to BIGEVOX10, a step is seen at *ca*. 500 °C on heating, corresponding to the β → γ transition, consistent with the thermal variation of lattice parameter plot (Figure 2d). However, no evidence was seen of an intermediate step that could be associated with an ε-phase in the Sn substituted system. High conductivity is achieved in the γ-phases of both systems. At 600 °C, the conductivity is 1.2 × 10^–1^ S cm^–1^ for BIGEVOX10 and 1.6 × 10^–1^ S cm^–1^ for BISNVOX05, with corresponding activation energy values of 0.33(2) eV and 0.21(2) eV, respectively; while at 300 °C, the conductivities for these two compositions are 3.0 × 10^–6^ S cm^–1^ and 2.0 × 10^–4^ S cm^–1^, with calculated activation energies of 1.39(7) and 0.99(1) eV, respectively. Therefore, despite BIGEVOX10 having a higher nominal vacancy concentration than BISNVOX05, it generally shows lower conductivity. In the case of the conductivity at 300 °C, this is readily explained by the fact that in the Ge system the more poorly conducting α-phase is present at this temperature, while in BISNVOX05 the system has transformed to the more highly conducting β-phase. At 600 °C, when both systems are in the fully disordered γ-phase, the extent of defect trapping by the substituent cations plays the dominant role in determining the conductivity.

### 3.3 Crystallographic Analysis

#### 3.3.1 α-Phase

**Figure 4:**
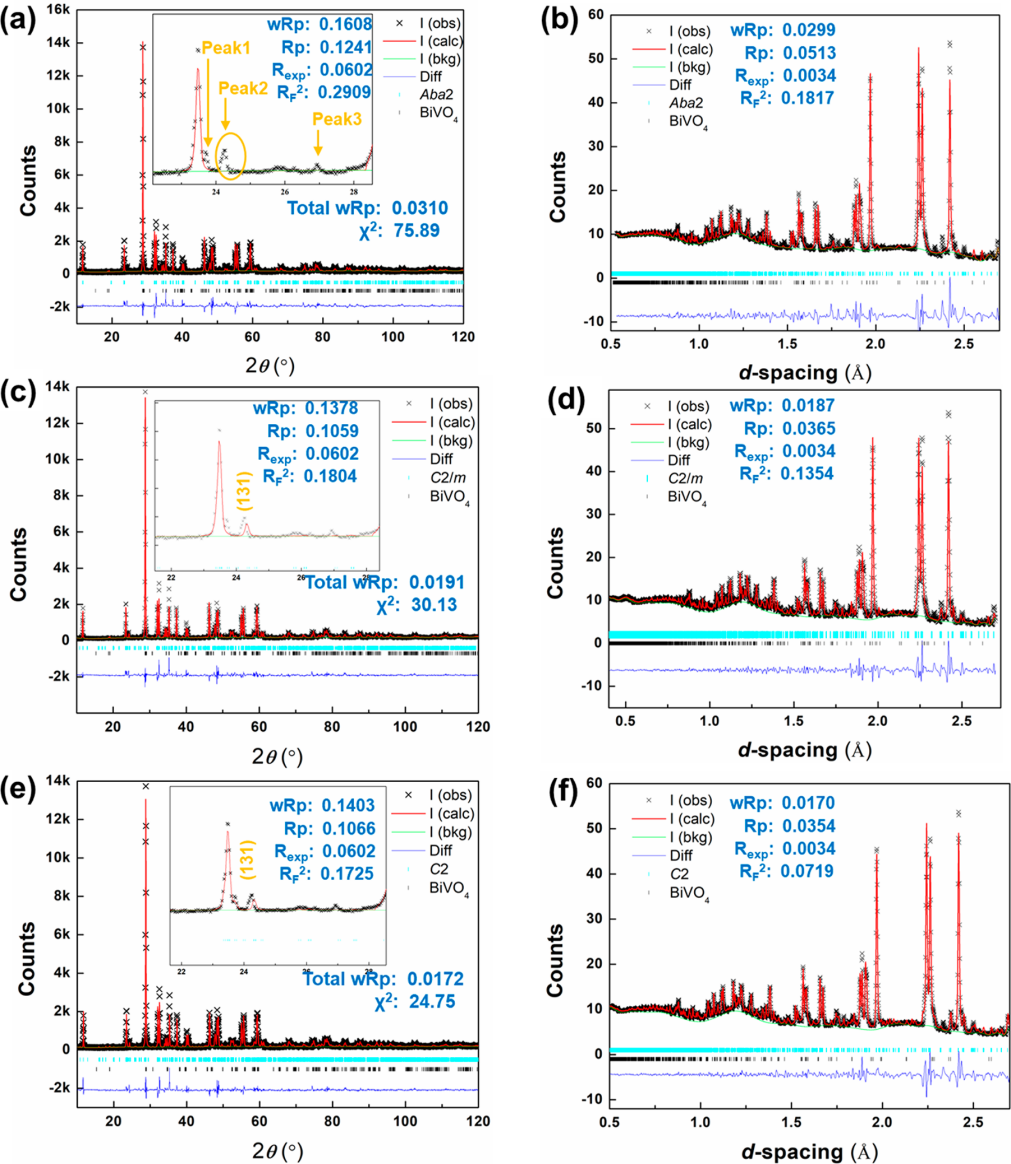
Fitted diffraction profiles showing fits to (a, c, e) X-ray and (b, d, f) neutron data for BIGEVOX10 at 25 °C using (a and b) *Aba*2 (c and d) *C*2/*m* and (e and f) *C*2 models. Magnifications of the X-ray fits are inset.

Fitted X-ray and neutron diffraction (bank 5) profiles for BIGEVOX10 at 25 °C are shown in Figure 4. As discussed above, the X-ray diffraction data for BIGEVOX10 show little evidence of the monoclinic distortion known to occur in the unsubstituted parent compound at room temperature. Nevertheless, to assess the true symmetry both monoclinic and orthorhombic models were analyzed. Three crystallographic models, in space groups *Aba*2 (*a* = 5.598 Å, *b* = 15.292 Å, *c* = 5.532 Å),^19^*C*2/*m*,^20^ and *C*2 (transformed from *A*2),^13^ were compared in the refinement. The inset images in Figure 4a,c,e show the fitting area between 22° and 28° (2θ) using the three models. A set of peaks at 23.53° (peak 1), 24.08° (peak 2), and 26.7° (peak 3) cannot be fitted using the *Aba*2 mean cell model. To index these reflections on a superlattice of the *Aba*2 model, the four-integer indexation method proposed by De Wolff^51^ was applied:
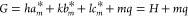
2
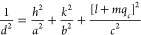
3where *G* is the reciprocal lattice vector, *m* is an integer, *q*_*c*_ is the modulation wave vector along *c*_m_, and *H* is any basic reciprocal lattice vector; *q*_*c*_ is expressed as 1/*p*_*c*_, where *p*_*c*_ is the modulation period. The indexing results are summarized in Table S2. Peak 1 can be indexed as (130), which breaks the general systematic absence condition caused by *A*-face centering in *Aba*2. Indexing peak 2 gives a modulation vector *q*_c_ of about 1/3, indicating the real structure is tripled along the *c-*axis in the *Aba2* model, corresponding to the (131) reflection in the *C*2/*m* and *C*2 models, similar to the 3*a*_m_ supercell for α-phase Bi_4_V_2_O_11_. Peak 3, which cannot be indexed in the *Aba*2 model, corresponds to the (005) reflection in the *C*2/*m* and *C*2 models. Therefore, the evidence suggests that the α-phase of BIGEVOX10 exhibits monoclinic rather than orthorhombic symmetry.

Despite the *R*_wp_ and *R*_p_ values for the BIGEVOX10 X-ray data being slightly higher for the *C*2 fit than the corresponding values for the *C*2/*m* fit, all other *R*-factors including the total *R* factors over all data sets and χ^2^ values were lower in the *C*2 model (Figure 4c–f, Tables 1 and S3). Bearing in mind the increased number of parameters in the *C*2 model, a statistical test of significance was performed using the method of Hamilton.^52^ The results in Table S4 confirm a significant improvement in fit using the *C*2 model compared with the *C*2/*m* model for BIGEVOX10 at the 99.5% confidence level. Similar conclusions were made comparing the *C*2 and *C*2/*m* models in BISNVOX05 (Figure S4 and Tables 1, S3, and S4). Though the 3*a*_m_ supercell model in space group *C*2 does not entirely describe all the superlattice peaks, it is considered to be the most satisfactory model. The refined structure and refinement parameters for BIGEVOX10 and BISNVOX05 at 25 °C using the *C*2 model are summarized in Table 1, with the refined atomic parameters shown in Tables S5 and S6 and selected bond lengths listed in Tables S7 and S8.

**Table 1:** Crystal and Refinement Parameters for BIGEVOX10 and BISNVOX05 at 25 °C

Sample Name		BIGEVOX10		BISNVOX05
Temperature (°C)		25 °C		25 °C
Chemical formula		Bi_2_V_0.9_Ge_0.1_O_5.45_	BiVO_4_	Bi_2_V_0.95_Sn_0.05_O_5.475_
Crystal system		*C*2	*I*2/*c*	*C*2
Lattice parameters (Å)		*a* = 5.6031(2)	*a* = 5.225(2)	*a* = 5.6057(2)
		*b* = 15.3214(5)	*b* = 11.696(4)	*b* = 15.3490(4)
		*c* = 16.5805(5)	*c* = 5.162(2)	*c* = 16.6134(5)
		β = 90.053(4)°	β = 90.28(2)	β = 90.038(4)°
Volume (Å^3^)		1423.39(13)	315.5(1)	1429.44(11)
*Z*		12	4	12
Phase fraction		97.02(7)%	2.98(1)%	100%
Density (calc) (g cm^–3^)		7.815	6.818	7.805
*R*-factors	Neutron back scattering	*R*_wp_ = 0.0170		*R*_wp_ = 0.0134
		*R*_p_ = 0.0354		*R*_p_ = 0.0258
		*R*_ex_ = 0.0034		*R*_ex_ = 0.0035
		*R*_F_^2^ = 0.0719		*R*_F_^2^ = 0.0533
	Neutron 90°	*R*_wp_ = 0.0161		*R*_wp_ = 0.141
		*R*_p_ = 0.0262		*R*_p_ = 0.0220
		*R*_ex_ = 0.0022		*R*_ex_ = 0.0035
		*R*_F_^2^ = 0.0516		*R*_F_^2^ = 0.0797
	X-ray	*R*_wp_ = 0.1403		*R*_wp_ = 0.1295
		*R*_p_ = 0.1066		*R*_p_ = 0.0964
		*R*_ex_ = 0.0602		*R*_ex_ = 0.0559
		*R*_F_^2^ = 0.1725		*R*_F_^2^ = 0.1171
	Totals	*R*_wp_ = 0.0172		*R*_wp_ = 0.0147
		*R*_p_ = 0.0891		*R*_p_ = 0.0823
No. of variables		198		186
χ^2^		24.75		16.92
No. of profile points	Neut. (bs)	3790		3540
	(90 °C)	2089		2054
	X-ray	3440		3440

To further confirm the noncentrosymmetric structures of BIGEVOX10 and BISNVOX05 at room temperature, both theoretical and experimental analyses were performed. First, molecular dynamics simulations were carried out for BIGEVOX10 and BISNVOX05 compositions based on a centrosymmetric ideal model. The RMC structures were then used for the prediction of charges using a machine learning method as described in the Experimental Section. The obtained values were then analyzed to calculate the spontaneous-polarization (*P*_s_) value, and the results are shown in Table S9. The calculated polarization values are all less than 2 μC cm^–2^ along the *x*, *y*, and *z* directions, with those for BIGEVOX10 slightly greater than those for BISNVOX05, indicating that both structures are weakly polar compared to classical polar materials such as BaTiO_3_ for which the *P*_s_ ≈ 19 μC cm^–2^.^53^ Second, the frequency dependence of dielectric relative permittivity (ε_r_) was measured as a function of temperature for both compositions (Figure S5). In BIGEVOX10 (Figure S5a), at all frequencies, ε_r_ generally increases as the sample is heated, with two anomalies found at *ca*. 450 and 550 °C, corresponding to the α → β and β → γ phase transitions, consistent with the observations in the VT-XRD patterns (Figure 2a). The dielectric loss also generally increases with increasing temperature up to a maximum at *ca*. 420 °C, before showing a frequency dependent drop at *ca*. 450 °C. Close inspection of the low temperature loss data reveals a broad frequency dependent peak in the range from 90 to 160 °C. Similar peaks in the permittivity plot are seen in BISNVOX05 (Figure S5b), corresponding to transition temperatures of 340 and 530 °C, again consistent with the transitions seen in the VT-XRD data (Figure S3a). The low temperature frequency dependent peak between 90 and 160 °C in the plots of the dielectric loss tangent for BISNVOX05 is much more evident than in the Ge system, particularly at low frequencies. Based on the observed results, it can be suggested that the temperature driven α → β and β → γ phase transitions correspond to weak ferroelectric (or ferrielectric) → weak ferroelectric, and weak ferroelectric (or ferrielectric) → paraelectric transitions. The low temperature frequency dependent loss peak is not accompanied by a major structural change on the crystallographic scale. This suggests a more subtle transition, possibly involving the formation of polar nanoregions as seen in relaxor ferroelectrics.^54^ Based on the crystallographic and electrical results, the α-phase of BIGEVOX and BISNVOX is suggested to be weakly polar and best described in space group *C*2.

**Figure 5:**
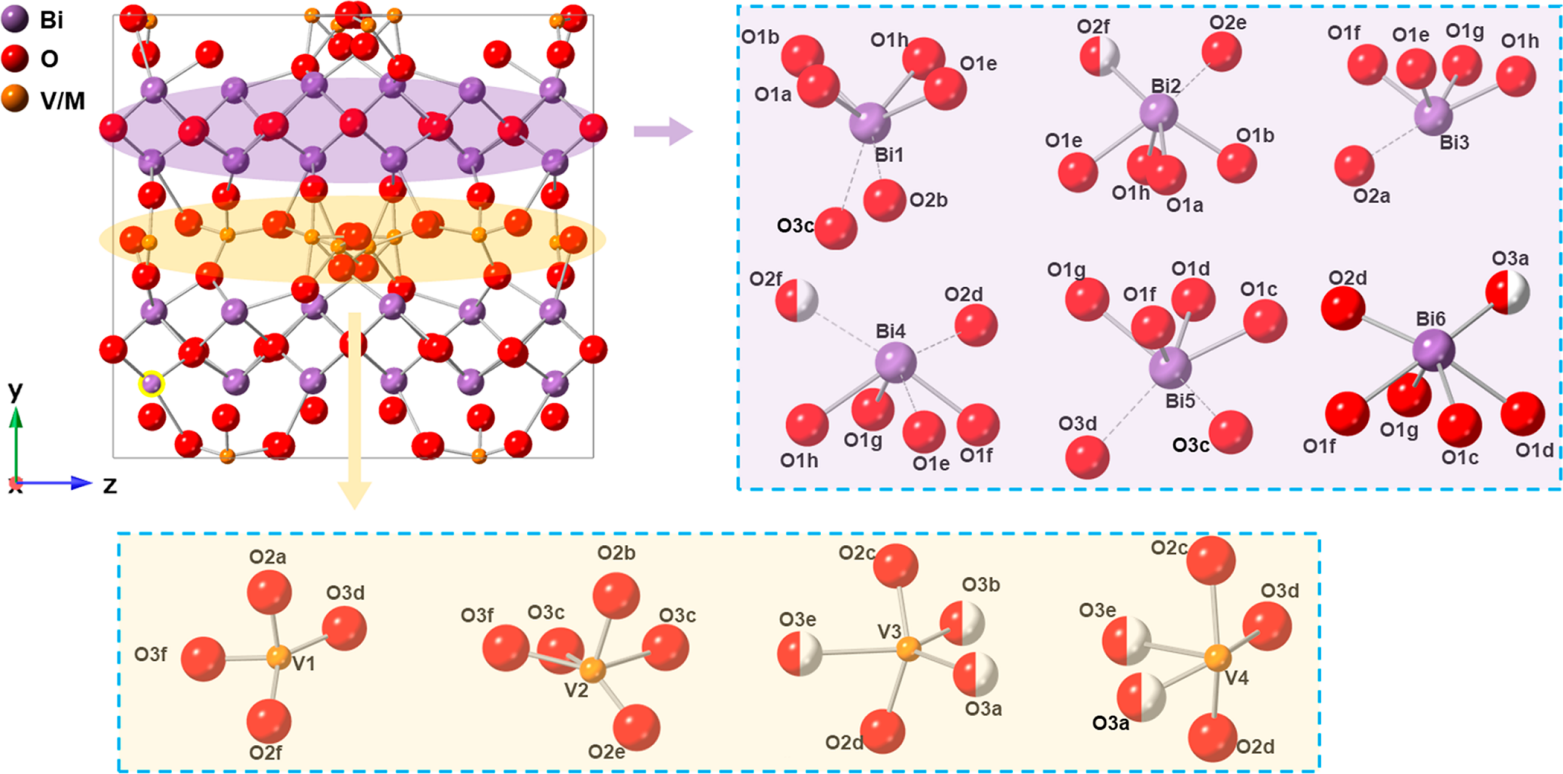
Refined crystal structure of α-BIGEVOX10 and local geometries of Bi atoms and M (V/Ge) atoms.

Figure 5 shows the refined structure for α-BIGEVOX10 at room temperature. Six crystallographically distinct Bi sites (Bi1–Bi6) are observed in the bismuthate layer. All the Bi–O(1) pairs (except Bi4–O(1e)) show significantly shorter contact distances than the sum of the ionic radii for Bi^3+^ and O^2–^ of 2.52 Å (assuming 8-coordination for Bi^3+^ and 2-coordination for O^2–^ ^55^) and can be considered as covalent bonds. Some short contacts are also observed between Bi and O atoms in the vanadate layer such as Bi2–O(2f) (2.368 Å), Bi6–O(2d) (2.269 Å), and Bi6–O(3a) (2.363 Å), with other Bi–O(2) and Bi–O(3) pairs showing longer contacts above 2.54 Å (Table S7). The shorter Bi–O(2) and Bi–O(3) contacts indicate greater covalency in the interaction between the vanadate and bismuthate layers. Similar covalent interactions are seen between the bismuthate and vanadate layers in α-BISNVOX05, with that for Bi6–O(2d) as low as 2.083 Å. In the vanadate layer, M (V/Ge or V/Sn) atoms share four different crystallographic sites (M1, M2, M3, and M4), with the M3 and M4 sites split into neighboring positions that cannot be simultaneously occupied. In BIGEVOX10, the average bond lengths for M–O(2) and M–O(3) are *ca*. 1.80 and 2.03 Å, respectively, giving an overall M–O average of 1.92 Å. Similar bond lengths are observed for BISNVOX05, with an average M–O bond length of 1.92 Å. Taking into account the partial occupancies of the O atom sites and assuming a cutoff distance of 2.5 Å, it is possible to calculate the average coordination numbers (*CN*) for each of the M sites in the vanadate layer. For BIGEVOX10, the calculated coordination numbers are M1 = 3.75, M2 = 5.0, M3 = 4.59, and M4 = 4.50, giving an average coordination number of 4.46 over all the M sites, while for BISNVOX05 the average coordination number is 4.82, with respective values of M1 = 4.925, M2 = 5.0, M3 = 4.425, and M4 = 4.925. It should be noted that the cutoff of 2.5 Å is significantly larger than the weighted sum of the ionic radii of M^*n*+^ and O^2–^ ions (assuming V, Ge, and Sn are 6-coordinate and O is 2-coordinate),^55^ as well as that for the covalent radii of 2.16 Å.^56^

#### 3.3.2 γ-Phase

**Figure 6:**
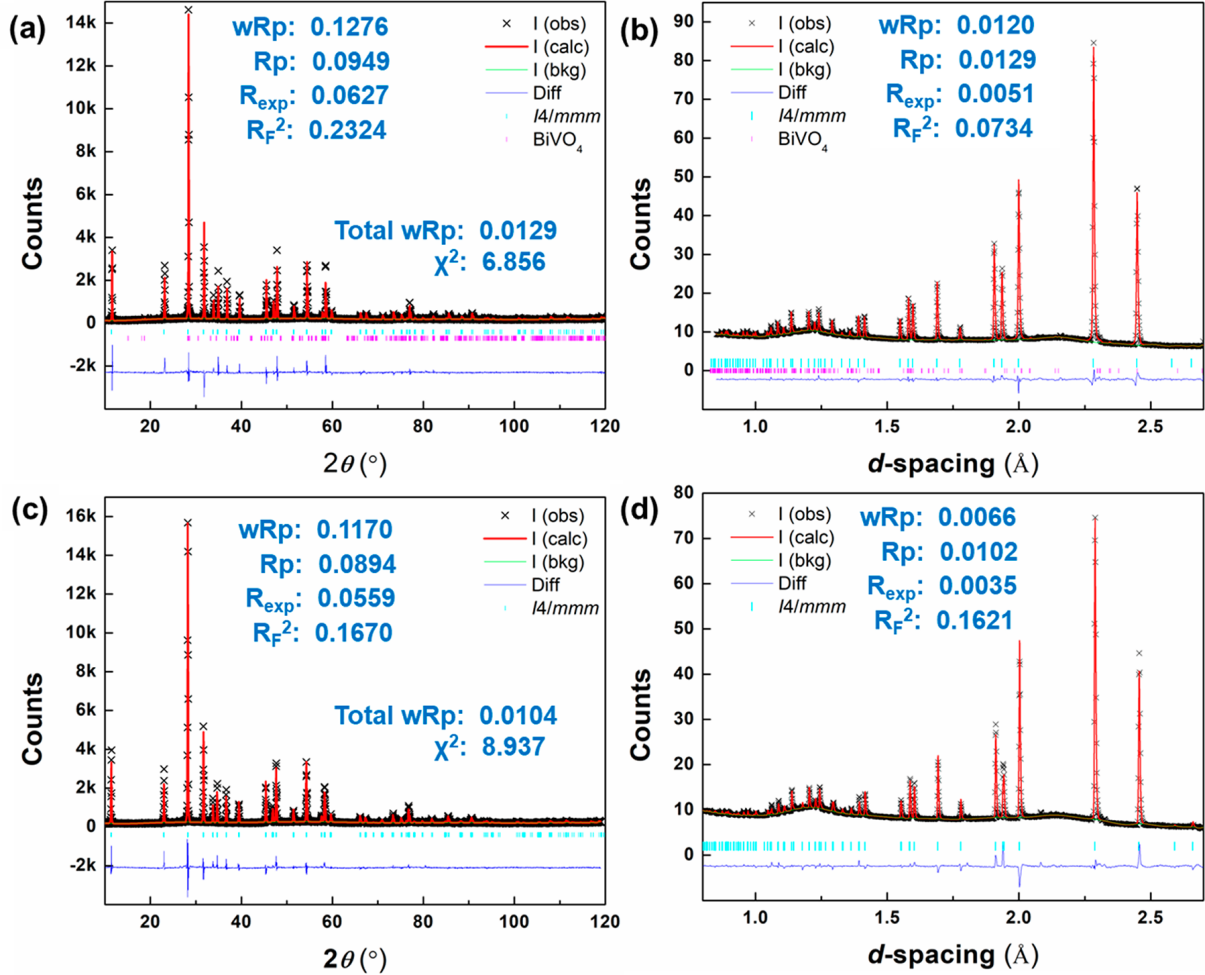
Fitted diffraction profiles showing fits to (a and c) X-ray and (b and d) neutron diffraction patterns for (a and b) BIGEVOX10 and (c and d) BISNVOX05 at 700 °C in space group *I*4/*mmm*.

Figure 6a,b shows X-ray and neutron (bank 5) profiles for BIGEVOX10 at 700 °C, fitted using tetragonal models in space group *I*4/*mmm*. The fitted X-ray and neutron profiles for BISNVOX05 at 700 °C are shown in Figure 6c,d, with the corresponding crystal and refinement parameters listed in Table 2, the refined atomic parameters in Table S10, and selected significant bond lengths given in Table S11. All the diffraction patterns were well fitted using the tetragonal models.

**Table 2:** Crystal and Refinement Parameters for BIGEVOX10 and BISNVOX05 at 700 °C

Sample Name		BIGEVOX10	BISNVOX05
Temperature (°C)		700 °C	700 °C
Chemical formula		Bi_2_V_0.9_Ge_0.1_O_5.45_	Bi_2_V_0.95_Sn_0.05_O_5.475_
Crystal system		*I*4/*mmm*	*I*4/*mmm*
Lattice parameters (Å)		*a* = 3.99553(6)	*a* = 3.9968(1)
		*c* = 15.4768(3)	*c* = 15.5209(5)
Volume (Å^3^)		247.077(10)	247.933(25)
Z		2	2
Phase fraction		99.600(9)%	100%
Density (calc) (g cm^–3^)		7.504	7.500
*R*-factors	Neutron (back scattering)	*R*_wp_ = 0.0120	*R*_wp_ = 0.0066
		*R*_p_ = 0.0129	*R*_p_ = 0.0102
		*R*_ex_ = 0.0051	*R*_ex_ = 0.0035
		*R*_F_^2^ = 0.0734	*R*_F_^2^ = 0.1621
	Neutron (90°)	*R*_wp_ = 0.0103	*R*_wp_ = 0.0113
		*R*_p_ = 0.0116	*R*_p_ = 0.0155
		*R*_ex_ = 0.0027	*R*_ex_ = 0.0023
		*R*_F_^2^ = 0.0760	*R*_F_^2^ = 0.4870
	X-ray	*R*_wp_ = 0.1276	*R*_wp_ = 0.1170
		*R*_p_ = 0.0949	*R*_p_ = 0.0894
		*R*_ex_ = 0.0627	*R*_ex_ = 0.0559
		*R*_F_^2^ = 0.2324	*R*_F_^2^ = 0.1670
	Totals	*R*_wp_ = 0.0129	*R*_wp_ = 0.0104
		*R*_p_ = 0.0767	*R*_p_ = 0.0722
No. of variables		120	117
χ^2^		6.856	8.937
No. of profile points	Neut. (bs)	2307	3539
	(90 °C)	1468	2161
	X-ray	3440	3411

**Figure 7:**
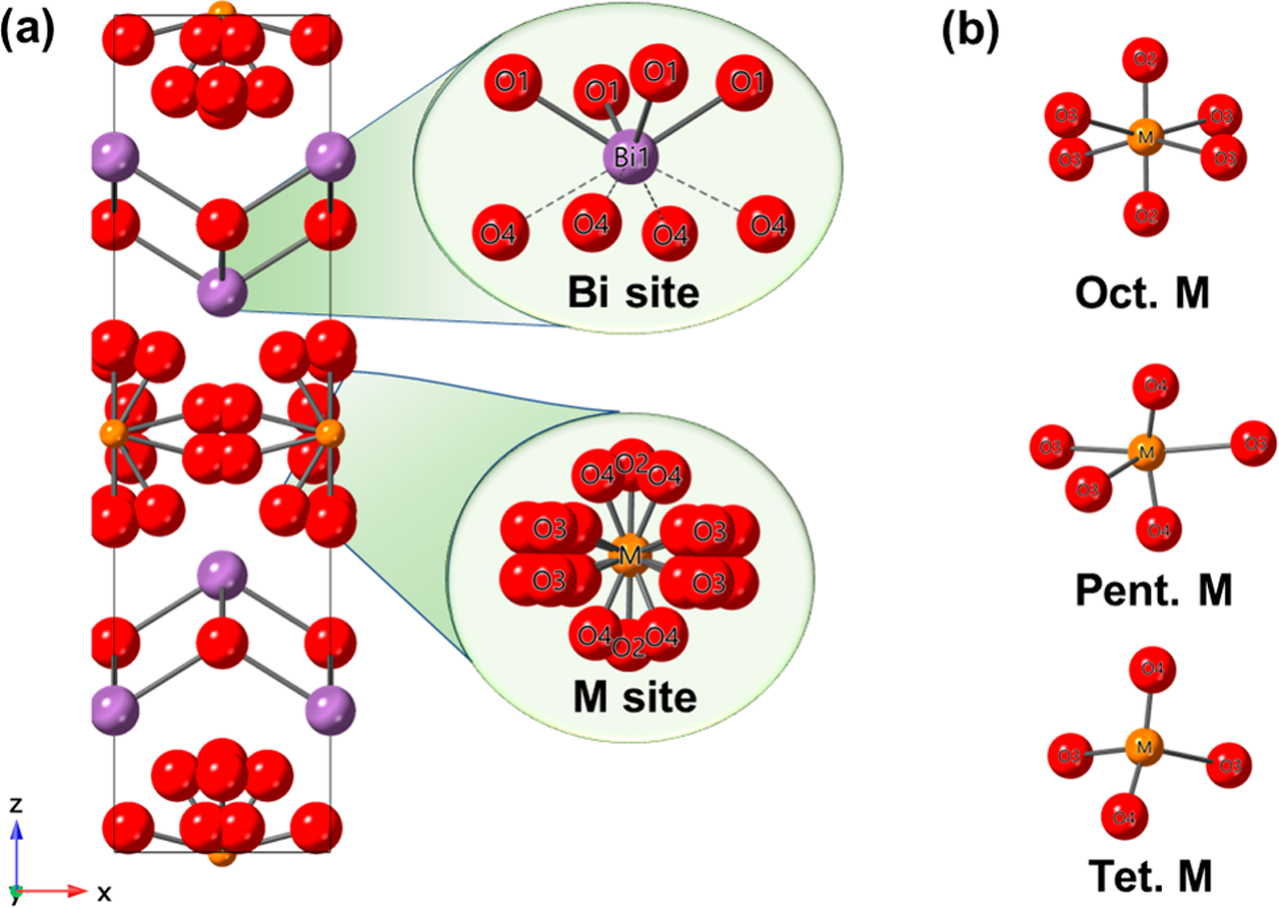
(a) Refined average γ-phase structure for BIGEVOX10 at 700 °C derived from Rietveld analysis and (b) the local geometries for ME (ME = V/Ge) atoms in the vanadate layer.

A representative image of the refined crystal structure for γ-BIGEVOX10 at 700 °C is shown in Figure 7a. In this structure, only single crystallographically distinct sites are seen for Bi and V/Ge. Two types of Bi–O contact are seen: shorter bonds (*ca*. 2.35 Å) to four O(1) atoms, in a pyramidal geometry, and longer contacts (≥2.60 Å) to four O(4) atoms. BISNVOX05 shows a similar γ-phase structure to BIGEVOX10 at 700 °C, with only one crystallographically distinct V/Sn site. For both compositions, refinements show the summed O(2) and O(4) contents are close to 2 per V/M site; thus the nonbridging apical positions can be considered as fully occupied, with all oxygen vacancies located in bridging equatorial positions, i.e., the O(3) sites, consistent with the EV model. Thus, the equatorial oxygen vacancy concentration can be obtained directly from the solid solution formula as 0.5 + *x*/2 per M atom and is equal to 0.55 and 0.525 for BIGEVOX10 and BISNVOX05, respectively. The theoretical average coordination number (*CN*) for M atoms in these two systems can also be calculated as the sum of the two apical oxygen atoms and those in the O(3) sites. Since each O(3) bridges two M atoms, then *CN* = 2 + 2 × *n*_O(3)_ (where *n*_O(3)_ is the number of O(3) atoms per M atom) and is equal to 4.9 and 4.95 for BIGEVOX10 and BISNVOX05, respectively.

As we have shown previously in other BIMEVOX systems,^57^ based on the O atom positions around the M atoms and their site occupancies it is possible to construct various coordination polyhedra around the M atoms (Figure 7b). Of these, octahedra could be formed using O(2) and O(3) atoms, while tetrahedra would involve O(4) and O(3) atoms. Starting with an initial assumption that only tetrahedral and octahedral geometries for M atoms in the vanadate layer are present, then O(2) and O(4) atoms would be exclusively associated with octahedra and tetrahedra, respectively. The fractions (*f*) of each polyhedral type can then be calculated from the site occupancies in Table S10 as
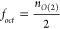
4
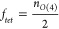
5For BIGEVOX10, the calculated values are *f*_oct_ = 0.382 and *f*_tet_ = 0.62. Since O(3) is common to both coordination polyhedra, the amount of O(3) required to satisfy the requirements of both types of polyhedra *n*_O(3)_ = 2*f*_oct_ + *f*_tet_ = 2 × 0.38 + 0.62 = 1.38. This is significantly lower than the total O(3) content derived from the values in Table S10 of 1.45 (0.0906 × 32/2) per V/Ge atom. This suggests that at 700 °C the polyhedral environment in the vanadate layer is more complicated and that the initial assumption of only octahedral and tetrahedral coordination polyhedra is flawed. Based on the known chemistry of V and Ge, coordination numbers greater than 6 or lower than 4 are unlikely, but a coordination number of 5 is possible. The excess of 0.07 O(3) atoms per metal atom would allow for 5-coordinate M atoms involving three bridging O(3) and two nonbridging O(4) atoms, as shown in Figure 7. A series of relations may then be constructed:
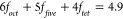
6
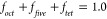
7
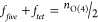
8Using simultaneous equations, the calculated polyhedral fractions for BIGEVOX10 at 700 °C are *f*_oct_ = 0.38, *f*_five_ = 0.14, and *f*_tet_ = 0.48. A similar analysis can be performed for BISNVOX05 and gives calculated polyhedral fractions of *f*_oct_ = 0.411, *f*_five_ = 0.128, and *f*_tet_ = 0.461. Thus, in both compositions at high temperatures, the average structure analysis suggests tetrahedral coordination geometries are dominant in the vanadate layer.

### 3.4 Local Structure Analysis

Representative fitted neutron *S*(*Q*), X-ray *F*(*Q*) and *G*(*r*) profiles, along with a final RMC configuration for BIGEVOX10 at 25 and 700 °C are shown in Figure S6a–d and S7a–d, respectively, with similar representative fitted data sets and final configurations for BISNVOX05 at 25 and 700 °C given in Figures S8a–d and S9a–d, respectively. It is seen that reasonable fits are achieved throughout, and the layered structure is maintained well at both temperatures for both compositions after the calculations.

**Figure 8:**
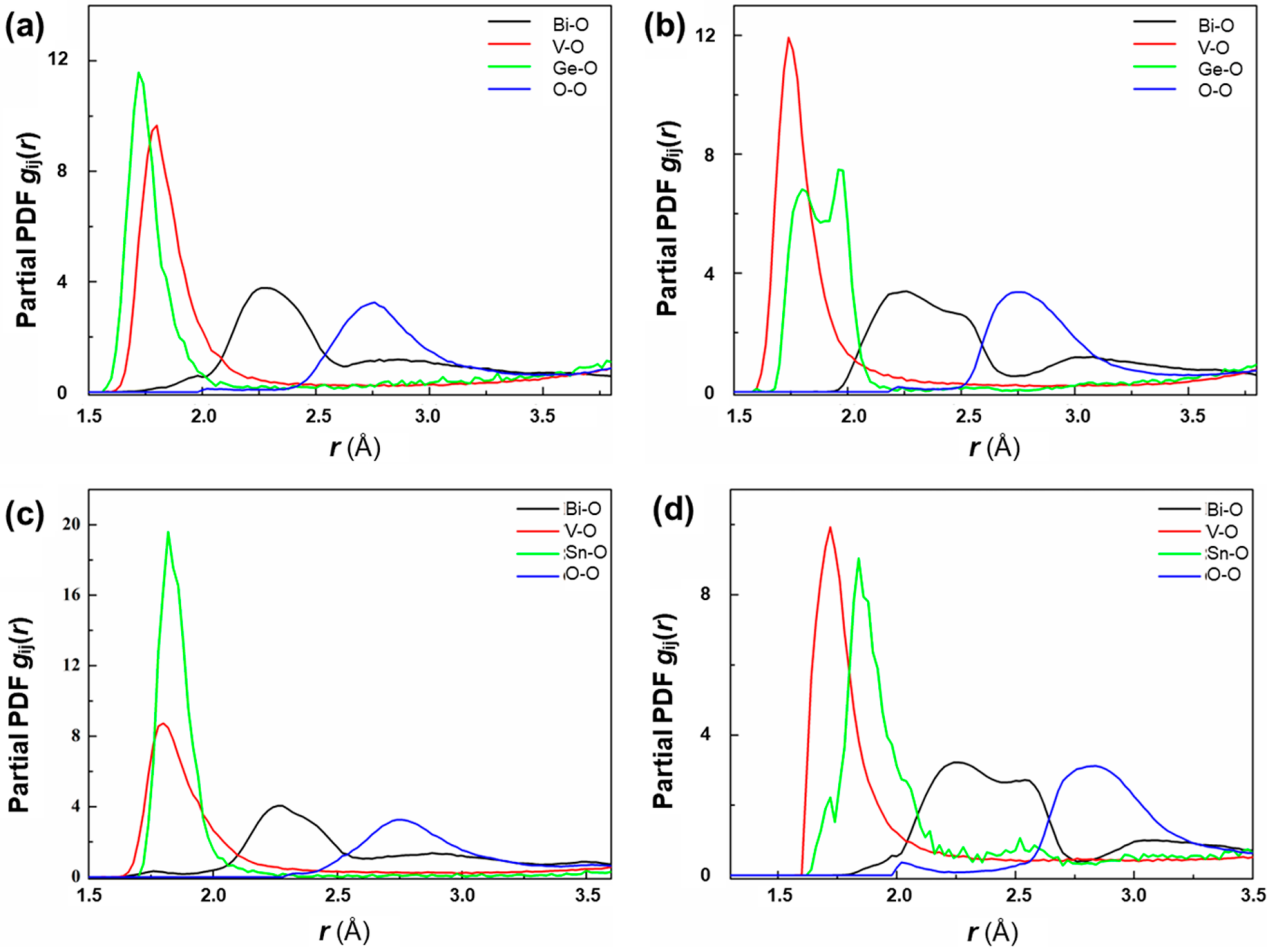
Selected M–O and O–O partial PDFs for (a and b) BIGEVOX10 and (c and d) BISNVOX05 at (a and c) 25 °C and (b and d) 700 °C. Each partial is derived from the average of 10 parallel sets of calculations.

The partial pair distribution functions (PDFs) for M–O and O–O correlations for BIGEVOX10 at 25 and 700 °C are shown in Figure 8a,b, while those for BISNVOX05 are shown in Figure 8c,d. At 25 °C, similar distributions are observed in the two compositions. The mean and modal contact distances for Bi–O, M–O, and O–O correlations are summarized in Table 3. At room temperature, the V–O correlation in BISNVOX05 shows a smaller modal distance of 1.77 Å compared with a value of 1.79 Å in BIGEVOX10. The modal distance for Sn–O (1.813 Å) is larger than that for Ge–O (1.72 Å) due to the larger ionic radius of Sn^4+^ (*r* = 0.69 and 0.53 Å for Sn^4+^ and Ge^4+^ in octahedral geometry^55^). In BIGEVOX10, the Bi–O partial PDF shows a modal peak centered at around 2.27 Å, with a second broad correlation at around 2.8 Å, corresponding to the short Bi–O(1) and long Bi–O(2) bonds in the crystallographic models. This is typical for Bi–O and reflects the asymmetric coordination environment of Bi due to stereochemical activity of the Bi 6*s*^2^ lone pair of electrons. At 700 °C, the Ge–O correlation is split into two peaks centered at 1.78 and 1.96 Å, which probably correspond to nonbridging and bridging Ge–O bonds, respectively. For both compositions, the main Bi–O peak at 700 °C appears to be more asymmetric than at room temperature, with the evolution of a discernible peak at around 2.6 Å, replacing the broad correlation at around 2.8 Å seen at room temperature. This suggests a shortening of the Bi–O contacts between the bismuthate and vanadate layers. At both studied temperatures, the V–O and M–O pairs in BIGEVOX10 show shorter mean contact distances than those in BISNVOX05. This is consistent with the weighted average values obtained from the crystallographic models (V/Ge–O: 1.91 Å vs V/Sn–O: 1.92 Å at 25 °C and V/Ge–O: 1.895 Å vs V/Sn–O: 1.898 Å at 700 °C) based on the data in Tables S5–S8.

**Table 3:** Selected Mean and Modal Contact Distances of Nearest Neighbor Atom Pairs in BIGEVOX10 and BISNVOX05 at the Studied Temperatures

		25 °C		700 °C	
Compositions	Type	Mean dist. (Å)	Modal dist. (Å)	Mean dist. (Å)	Modal dist. (Å)
BIGEVOX10	Bi–O	2.315(1)	2.265(4)	2.343(1)	2.192(4)
	V–O	1.881(2)	1.79(10)	1.838(1)	1.78(1)
	Ge–O	1.788(6)	1.72(1)	1.897(3)	1.83(2)
	O–O	2.898(1)	2.728(6)	2.920(2)	2.718(3)
BISNVOX05	Bi–O	2.361(1)	2.266(2)	2.370(2)	2.202(4)
	V–O	1.913(2)	1.768(8)	1.844(3)	1.673(4)
	Sn–O	1.867(6)	1.813(10)	1.952(9)	1.895(21)
	O–O	2.899(1)	2.744(5)	2.945(1)	2.812(4)

**Figure 9:**
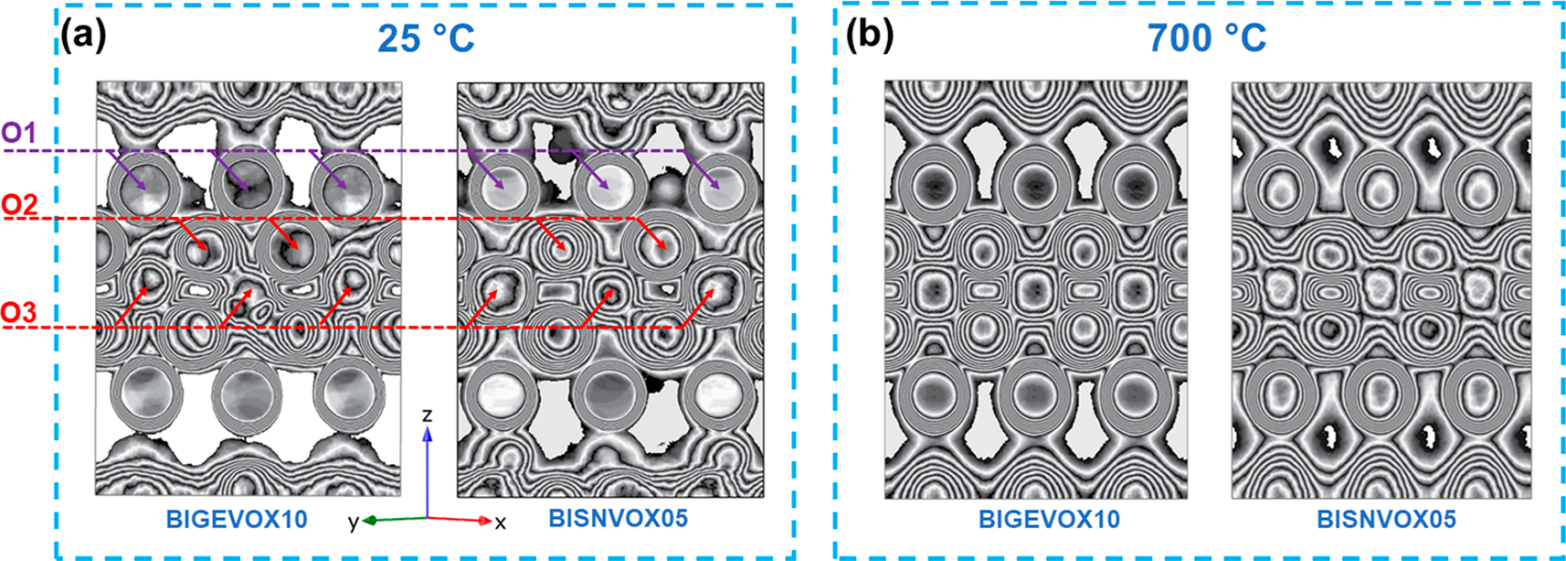
(a and b) Oxygen density in the equivalent (110) mean cell plane for BIGEVOX10 and BISNVOX05 compositions, at (a) 25 °C and (b) 700 °C. Plots were derived from final RMC configurations folded back into a single mean cell.

Figure 9a,b shows a comparison of oxygen number density in the (110) plane of the mean cell for the two compositions at 25 and 700 °C derived from folding the final RMC configuration into a single crystallographic unit cell. It is seen that the oxygen density in the bismuthate layer is well-defined, while that in the vanadate layer is much more diffuse between both apical and equatorial sites. At 700 °C, the oxygen distribution shows higher symmetry than at room temperature, reflecting the disordered character of the γ-phase. No significant difference can be observed between BIGEVOX10 and BISNVOX05 at the same temperature, indicating similar average structures for these two compositions.

**Figure 10:**
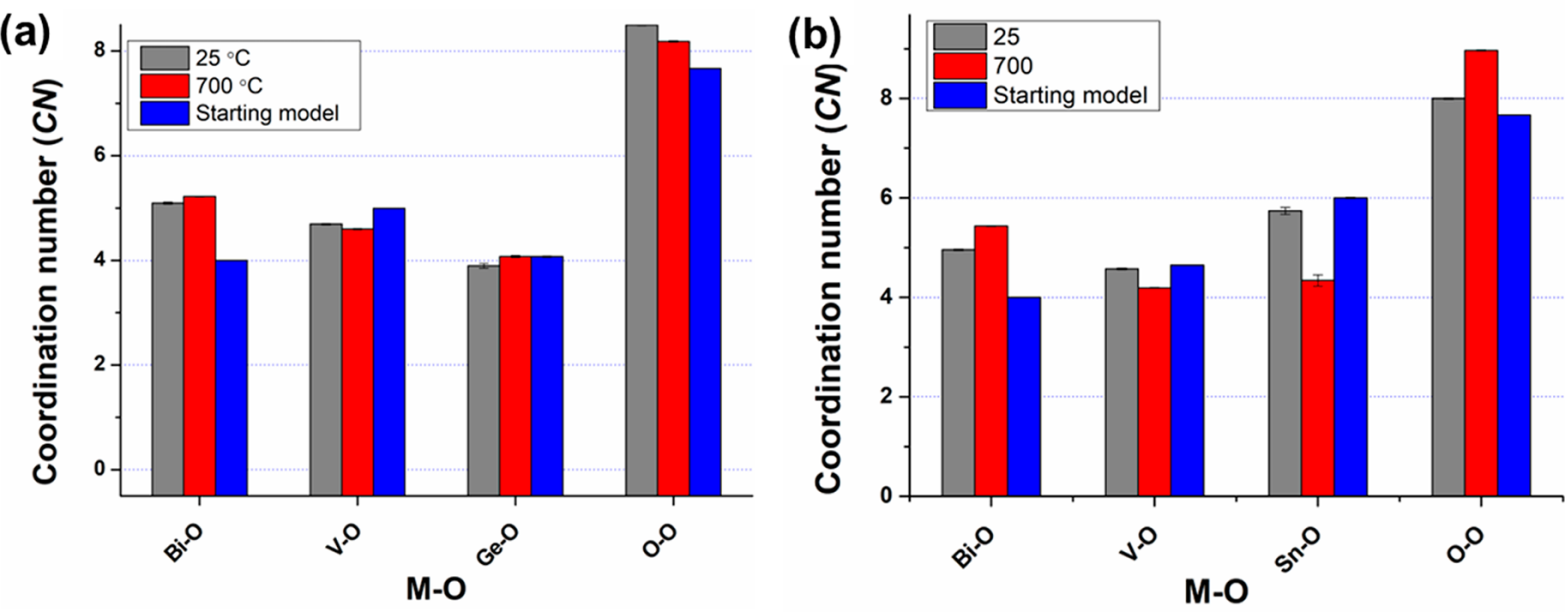
Coordination numbers for metal and oxygen atoms at room temperature and 700 °C derived from the final RMC configurations for (a) BIGEVOX10 and (b) BISNVOX05.

Figure 10 compares the *CN*s for Bi and M (M = V, Ge, Sn) in BIGEVOX10 and BISNVOX05 at the two studied temperatures. The Bi–O *CN*s show a significant increase from 4 in the starting model to around 5 in the final configurations for both compositions at both studied temperatures. This suggests a much stronger interaction between the bismuthate and vanadate layers than that in the starting models which were based on the idealized structure. This is consistent with the observed relatively short bond lengths between Bi and some O atoms in the vanadate layer in the average structure analysis (Tables S7, S8, and S11). At 25 °C, the *CN* for V in both compositions is close to 4.5, while the value for Ge is approximately 4.0, compared to a value of *ca*. 6 for Sn, consistent with predominantly tetrahedral and octahedral geometries, respectively. In BIGEVOX10 at 25 °C, the average *CN* over all V/Ge sites derived from the RMC analysis is 4.51, slightly higher than the value of 4.43 obtained in the crystal structure analysis. Similarly, in BISNVOX05 at room temperature, the RMC model derived *CN* over all V/Sn sites is 4.63, being slightly higher than the value of 4.52 derived from the crystallographic analysis. These small discrepancies highlight the deficiency in the description of local structure using the average crystallographic model. At 700 °C, the V *CN*s for both compositions show an apparent drop compared to those observed at 25 °C. Interestingly, both the Ge and the Sn *CNs* approach 4 at 700 °C, with that for Ge increasing slightly to 4.0 and that for Sn showing a significant drop to 4.3 compared to the room temperature values.

Breakdowns of the *CN* distributions for V/Ge in BIGEVOX10 and V/Sn in BISNVOX05 with O at the two studied temperatures are summarized in Table 4. For both compositions, V atoms are mainly in four- and five-coordinate geometries with lesser amounts of six-coordinate geometry. In BIGEVOX10, Ge shows predominantly four-coordinate geometry at both 25 and 700 °C. This situation is different for Sn, where at room temperature most Sn atoms (*ca*. 75%) adopt six-coordinate geometry, but the fraction drops significantly at 700 °C, where four-coordinate geometry becomes dominant. For both compositions, the polyhedral distributions obtained from the RMC analysis at 700 °C differ somewhat from those derived from the average structure analysis. In both methods the tetrahedral fraction represents the dominant coordination geometry for the two compositions at 700 °C. However, the average structure analysis shows significantly higher proportions of octahedra and lower proportions of pentacoordinated atoms than the RMC analysis. In the case of γ-BIGEVOX10, the average structure analysis concluded that 38% of all M atoms are octahedral, 14% pentacoordinated, and 48% tetrahedral compared with values of 11.6%, 32.5% and 46.4%, respectively from the RMC analysis. This apparent discrepancy can be explained by considering the assumption made in the average structure analysis that all equatorial O(3) atoms bridge two M atoms. The RMC analysis reveals that at the local level some equatorial atoms are significantly closer to one M atom center than the other, to the extent that the longer interaction can be considered to be nonbonding, hence lowering the average coordination number and reducing the fraction of six-coordinate M atoms. A similar phenomenon is observed in γ-BISNVOX05 at 700 °C, where the average structure analysis indicated percentages of 41.1% octahedral, 12.8% pentacoordinate, and 46.1% tetrahedral compared to the respective values of 4.7%, 25.4%, and 55.0% in the RMC analysis. It should be noted here that the coordination number distributions from the RMC analysis also include small percentages of unrealistic lower cation coordination numbers, reflecting the statistical nature of this analysis method.

**Table 4:** First-Shell Coordination Numbers (*CN*s) for V, Ge and Sn atoms in vanadate layer

			25 °C		700 °C	
	Cation	*CN*s	Percentage (%)	Ave.	Percentage (%)	Ave.
BIGEVOX10	V	1	0.0	4.59(1)	0.0	4.50(1)
		2	0.55(24)		0.48(13)	
		3	8.22(107)		8.95(29)	
		4	36.42(138)		42.90(129)	
		5	40.54(110)		34.87(138)	
		6	14.22(075)		12.78(81)	
	Ge	1	0.0	3.83(5)	0.0	4.02(2)
		2	0.56(61)		0.05(15)	
		3	23.15(444)		10.39(18)	
		4	70.09(558)		77.42(169)	
		5	5.37(184)		11.66(159)	
		6	0.84(50)		0.49(49)	
BISNVOX05	V	1	0.0	4.57(1)	0.0	4.19(1)
		2	0.47(25)		0.59 (23)	
		3	8.37(87)		14.46(101)	
		4	38.01(103)		55.30(124)	
		5	39.23(120)		25.07(101)	
		6	13.90(062)		4.54(49)	
	Sn	1	0.0	5.73(7)	0.0	4.34(9)
		2	0.0		0.29(45)	
		3	0.19(56)		11.86(246)	
		4	1.48(181)		49.02(663)	
		5	23.70(716)		30.69(498)	
		6	74.63(731)		8.14(278)	

**Figure 11:**
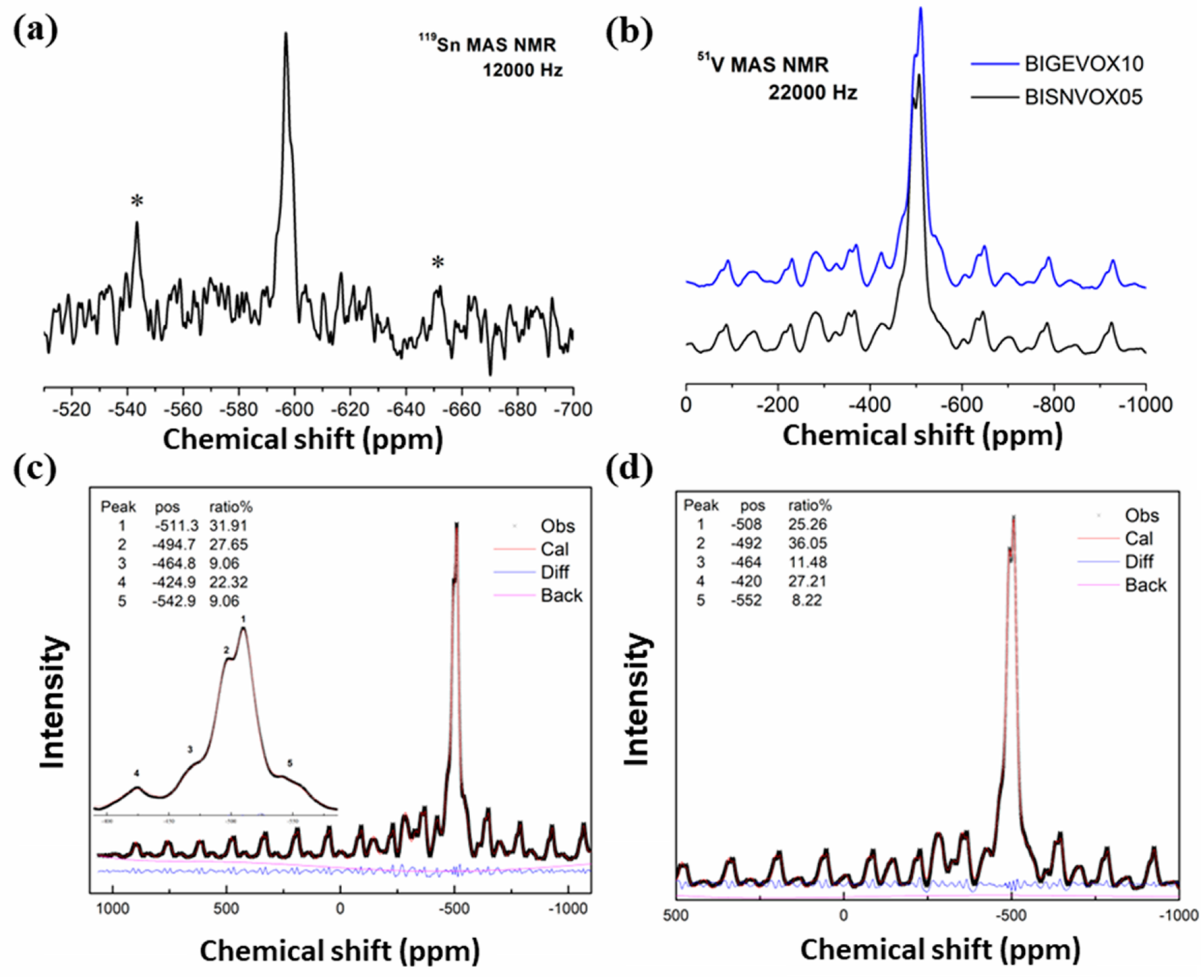
Solid state NMR spectra showing (a) ^117^Sn spectrum for BISNVOX05 and (b) comparison of ^51^V spectra for BIGEVOX10 and BISNVOX05; fitted ^51^V spectra for (c) BIGEVOX10 and (d) BISNVOX05.

The coordination environments of Sn and V were further analyzed through ^119^Sn and ^51^V MAS solid-state NMR. Figure 11a shows the ^119^Sn solid-state NMR spectrum of α-BISNVOX05 at room temperature and reveals an asymmetric peak centered at *ca*. −597 ppm flanked by spinning sidebands. This reflects the low symmetry of the α-phase, where the cations in the vanadate layer are located in a number of crystallographically distinct sites. The observed chemical shifts are close to that for octahedral Sn in SnO_2_ at −602 ppm,^58,59^ suggesting that Sn mainly adopts six coordination in α-BISNVOX05, consistent with the RMC analysis. Figure 11b shows the ^51^V solid-state NMR spectra for BIGEVOX10 and BISNVOX05 compositions at room temperature. In both compositions, the main resonance occurs at *ca*. −500 ppm and is consistent with the reported ^51^V spectrum for α-Bi_4_V_2_O_11_.^25,11^ The two α-phase compositions show similar V spectra, with similar resonance shift values. Figure 11c,d shows the fitted ^51^V NMR spectra for BIGEVOX10 and BISNVOX05, respectively. Five resonances are observed in each case. The one sited at *ca*. −425 ppm is assigned to tetrahedral vanadium,^25^ with the one at *ca*. −465 ppm assigned to distorted tetrahedral vanadium. Considering shielding effects, the resonance at *ca*. −495 ppm is assigned to pentacoordinate V, and the one at *ca*. −511 ppm is assigned to octahedral V.^11^ The resonance at −543 ppm is not seen in α-Bi_4_V_2_O_11_ and is attributed here to octahedral V with a substituent atom as a next-nearest neighbor. For BIGEVOX10, the fitted area fraction for all tetrahedral peaks (at −424 ppm and −464 ppm) is *ca*. 31%, comparable to the value observed in the RMC analysis (*ca*. 36% in Table 4), while for BISNVOX05 the summed value is *ca*. 39%, in good agreement with the value of 38.0% derived from the RMC calculations. The observed pentacoordinate fractions for BIGEVOX10 (*ca*. 28%) and BISNVOX05 (*ca*. 36%) are both closer to the RMC results than those from the average structure analysis.

Using the final RMC configurations, it is possible to analyze vacancy distributions around the M sites. A comparison of the vacancy concentration per M atom for BIGEVOX10 and BISNVOX05 at 25 and 700 °C is shown in Table 5. In the two systems, both equatorial and apical vacancies are found, with the former type dominant at the two studied temperatures. In BIGEVOX10, there are generally fewer vacancies around V atoms than Ge atoms, while in BISNVOX05, there are more vacancies around V atoms than Sn, consistent with the *CN* analysis in Table 4. At high temperature, an increase in apical vacancy fraction is seen for both compositions, with that for BISNVOX05 more significant, increasing from *ca*. 15% at 25 °C to *ca*. 30% at 700 °C.

**Table 5:** Equatorial Vacancy (EV) and Apical Vacancy (AV) Concentrations per M Atom (M = Ge/V) in the Starting and Final Configurations for BIGEVOX10 at 25 and 700 °C, along with Their Standard Deviations over 10 Parallel Configurations

		BIGEVOX10		BISNVOX05	
	Vacancy type	25 °C	700 °C	25 °C	700 °C
Final configuration	AV/M	0.040(05)	0.118(06)	0.081(10)	0.157(16)
	EV/M	0.510(05)	0.431(06)	0.444(10)	0.367(16)
	AV/V	0.039(08)	0.101(09)	0.085(12)	0.153(20)
	EV/V	0.485(09)	0.393(04)	0.469(13)	0.368(09)
	AV/Ge	0.056(19)	0.254(25)	0.006(09)	0.142(61)
	EV/Ge	0.834(40)	0.714(23)	0.047(19)	0.174(74)
Starting configuration	AV/M	0	0	0	0
	EV/M	0.550	0.550	0.525	0.525

**Figure 12:**
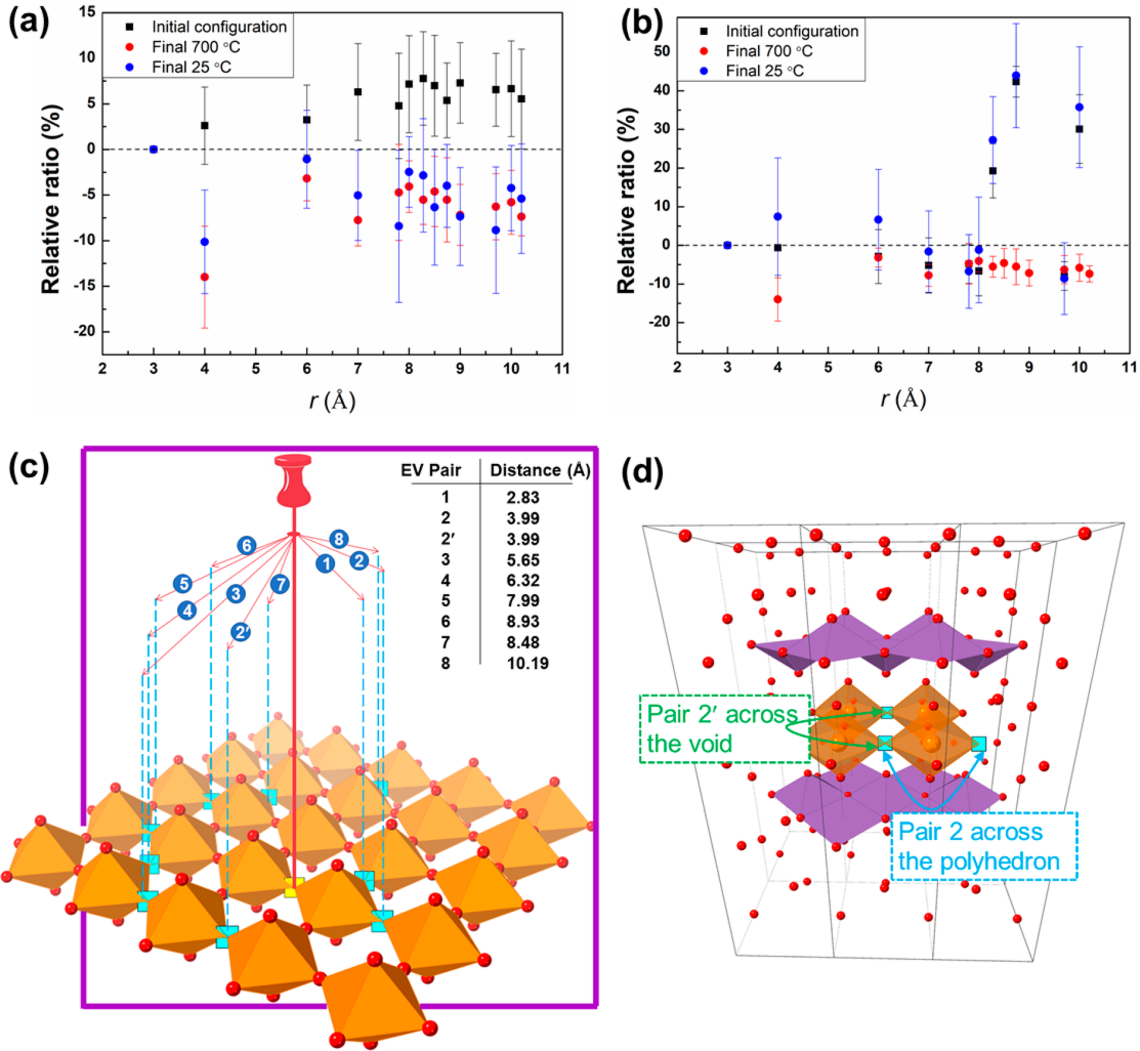
Radial variation of EV shell content expressed as a ratio with respect to the EV content of the first shell in (a) BIGEVOX10 and (b) BISNVOX05 at 25 and 700 °C; (c) a schematic map showing the vacancy pairs at different cutoff distances; and (d) a representative image showing the two distinct vacancy pairs in the next nearest shell.

To determine whether vacancy ordering is present, it is helpful to examine the radial distribution of equatorial vacancies around M atoms in the vanadate layer. Normalizing these numbers to the number of vacancies in the first coordination shell and subtracting the values for a random distribution at each shell gives a relative ratio as shown in Figure 12a,b for BIGEVOX10 and BISNVOX05, respectively. This allows for positive or negative deviations from the random distribution (represented by the dashed line at zero relative ratio) to be easily recognized. A schematic image showing the equatorial vacancy pairs at different cutoff distances is shown in Figure 12c. For BIGEVOX10, similar levels of deviation from the random model are observed, but all are negative. In most cases the differences are within the standard deviation of the random model. However, in the case of the next-nearest neighbor EV–EV correlation at *ca*. 4 Å a significant negative deviation from the random model is seen, indicating fewer vacancies occur at this distance than would be expected from a simply random distribution. This next-nearest neighbor correlation can occur either on the same polyhedron or on a neighboring one across the void between four corner sharing polyhedra (Figure 12d). Analysis of the models at the two temperatures indicates that the majority of the observed vacancy pairs at this distance are on neighboring polyhedra rather than the same polyhedron, suggesting that incorporation of two vacancies on the same polyhedron directly opposite each other is relatively unfavorable. This preferential ordering of EV pairs is formed along the ⟨100⟩ (or ⟨010⟩ in tetragonal symmetry) directions with the majority of EVs paired across the void between four V/Ge polyhedra. Interestingly, in BISNVOX05, the vacancy distribution at long distances (>8 Å) is far from random at 25 °C, being similar to that in the quasi-random starting model, while at shorter distances a more random distribution is seen. This is different from the observations for BIGEVOX10, where preferential ordering occurs at shorter distances. At 700 °C, there is a significant deficiency of vacancies in the second shell in the ⟨100⟩/⟨010⟩ directions, as observed in BIGEVOX10, while at longer distances the distributions are more random compared to the room-temperature data. These differences between BIGEVOX10 and BISNVOX05 at room temperature are likely due to the difference in preferred coordination geometry of the substituent cation, with the former predominantly tetrahedral and the latter predominantly octahedral at room temperature. At elevated temperatures, both systems show predominantly tetrahedral coordination geometry for the M atom and show similar vacancy distributions.

## 5 Conclusions

In this work, the long-range and local structures of two BIMEVOX compositions, BIGEVOX10 and BISNVOX05, were characterized. Both compositions show ordered α-phase structures in monoclinic symmetry at room temperature, with reversible α ↔ β and β ↔ γ phase transitions at elevated temperatures. The disordered γ-phase for both compositions at high temperature was characterized in tetragonal symmetry. Average structure analysis for both α-phase compositions shows four crystallographically distinct Bi and four V/ME sites, with the V/ME coordination number generally lower than theoretical values, while the high-temperature γ-phase shows only a single crystallographically distinct site for V/ME. Examination of the local structure through RMC analysis using both neutron and X-ray total scattering data at 25 and 700 °C reveals vanadium atoms adopt four, five, and six coordinate geometries in the α- and γ-phases of both compositions, as also evidenced by ^51^V solid state NMR analysis. Ge was found mainly to show tetrahedral geometry at the two studied temperatures, while Sn shows preferential octahedral geometry at 25 °C and tetrahedral geometry at 700 °C. The different coordination preferences for Ge and Sn lead to distinct oxygen and vacancy distributions in the vanadate layer in BIGEVOX10 and BISNVOX05. Oxygen vacancies are mainly found to be distributed in equatorial sites for both compositions at the two studied temperatures, with an increase in the concentration of apical vacancies at 700 °C. Vacancy ordering differs in these two compositions, with a nonrandom deficiency in vacancy pairs in the second-nearest shell for BIGEVOX10 along the ⟨100⟩ tetragonal direction and a long-distance (>8 Å) ordering of equatorial vacancies for BISNVOX05 at room temperature, which appears to be associated with the preferred octahedral geometry of Sn at this temperature. Both systems exhibit high conductivity when in the γ-phase, with values of 1.6 × 10^–1^ S cm^–1^ and 1.2 × 10^–1^ S cm^–1^ at 600 °C, for BISNVOX05 and BIGEVOX10, respectively, but conductivity drops significantly at lower temperatures when the ordered phases appear.

## Data Availability

Neutron data used in this work are available at https://doi.org/10.5286/ISIS.E.RB1820126.

## Supporting Information Available

Rietveld refinement procedure for the α- and the γ- phases, SEM images with EDX analysis, thermal evolution of X-ray and neutron diffraction patterns, fitted diffraction patterns, crystallographic parameters, permittivity results, and fitted neutron *S*(*Q*) and *G*(*r*) and X-ray *F*(*Q*) profiles (PDF)

## Accession Codes

CSD 2224495–2224499 contain the supplementary crystallographic data for this paper. These data can be obtained free of charge via www.ccdc.cam.ac.uk/data_request/cif, by emailing data_request@ccdc.cam.ac.uk, or by contacting The Cambridge Crystallographic Data Centre, 12 Union Road, Cambridge CB2 1EZ, U.K.; fax: + 44 1223 336033.
