# Supplementary material for: Local Structure in α-BIMEVOXes (ME = Ge, Sn)
Source: Chem Mater. 2022 Dec 22;35(1):189–206. doi: 10.1021/acs.chemmater.2c03001 (PMC9835824; doi:10.1021/acs.chemmater.2c03001)
Supplement: Supplementary file 1 — cm2c03001_si_001.pdf [file cm2c03001_si_001.pdf]

Supporting information for

Local structure in α-BIMEVOXes (ME = Ge, Sn)

Yajun Yue,[a, e] Aleksandra Dzięgielewska,[b] Man Zhang,[c] Stephen Hull,[d] Franciszek Krok,[b] Richard M. Whiteley,[c] Harold Toms,[a] Marcin Malys,[b] Xuankai Huang,[a] Marcin Krynski,[b] Ping Miao,[e] Haixue Yan,[c] and Isaac Abrahams[*a]

[a]Department of Chemistry, Queen Mary University of London, Mile End Road London, E1 4NS, U.K.

[b]Faculty of Physics, Warsaw University of Technology, ul. Koszykowa 75, 00-662 Warsaw, Poland.

[c]School of Engineering and Materials Science, Queen Mary University of London, Mile End Road London, E1 4NS, U.K.

[d]Science and Technology Facilities Council, ISIS Facility, Rutherford Appleton Laboratory, Chilton, Didcot, Oxon. OX11 OQX, U.K.

[e]Institute of High Energy Physics, Chinese Academy of Sciences, Beijing 100049, China.

Rietveld refinement process

The refinement process for the *C*2 model is described as follows: the background, zero corrections and peak shapes were refined first, followed by refinement of the thermal parameters. The isotropic thermal parameters for like atoms (Bi and O) were initially tied together in the refinement, while for V/Ge they were held at 0.02 $Å^2$. Subsequently, the thermal parameters for oxygen atoms were grouped into three types: bismuthate layer oxygen atoms (O(1a-h)), apical oxygen atoms (O(2a-f)) and equatorial oxygen atoms (O(3a-f)) and each group refined independently of other groups until convergence was achieved. Free refinement of the oxygen site occupancy parameters confirmed full occupation of O(1a-h) sites. For α-BIGEVOX10, short intersite contact distances between adjacent O(3a) and O(3e) prevent simultaneous occupation and therefore site occupancies of each were fixed at 0.5. All other O(2) and O(3) sites except for O(2f) and O3(b) were found close to 1.0 and thus a linear constraint was applied between O(2f) and O3(b) to refine their occupancies to maintain the calculated overall oxygen stoichiometry. For α-BISNVOX05, close contacts were among O(2d), O(3a) and O(3e) sites, thus their occupancies were fixed to be 0.5. Occupancies for all other O(2) and O(3) sites except for the O(3b) were found close to 1.0, so the residual stoichiometry-dependent oxygen vacancies were placed on the O(3b) site.

For the γ-phase structure refinements, isotropic thermal parameters were refined for all atoms with those for V and Ge, and O(2) and O(4) tied together. In this model, the oxygen atoms can be described as: O(1) in the 4*d* site in the bismuthate layer, O(2) and O(4) in apical positions in the vanadate layer, and O3 in an equatorial site in the vanadate layer. The total number of apical O (O2 and O4) per V/Ge was constrained to 2 and the O(3) site occupancy was fixed at the calculated value.

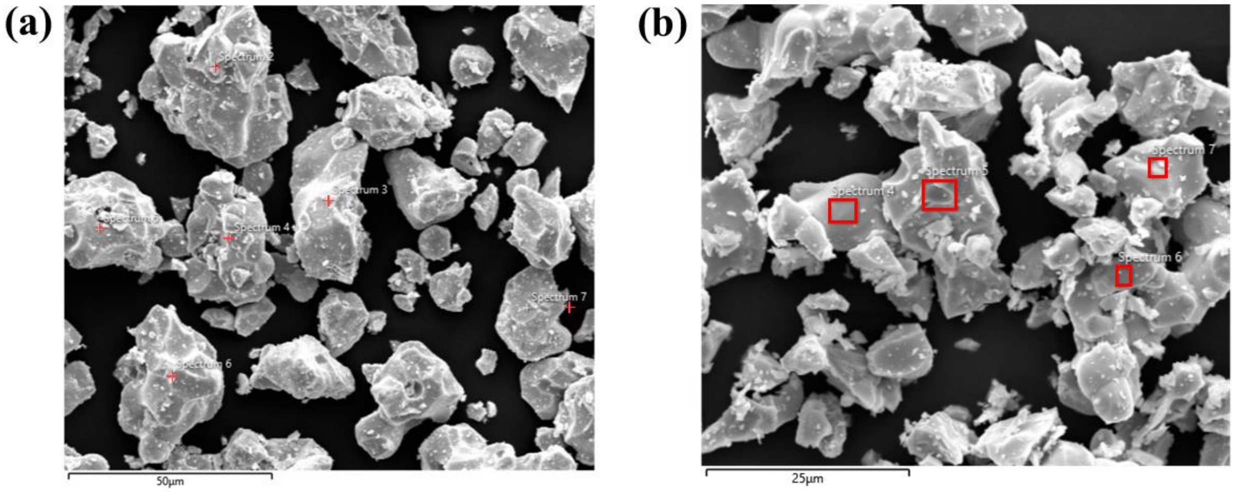

Fig. S1 SEM images of $Bi_2V_{0.90}Ge_{0.10}O_{5.45}$ and $Bi_2V_{0.95}Sn_{0.05}O_{5.475}$ powder used for EDX analysis.

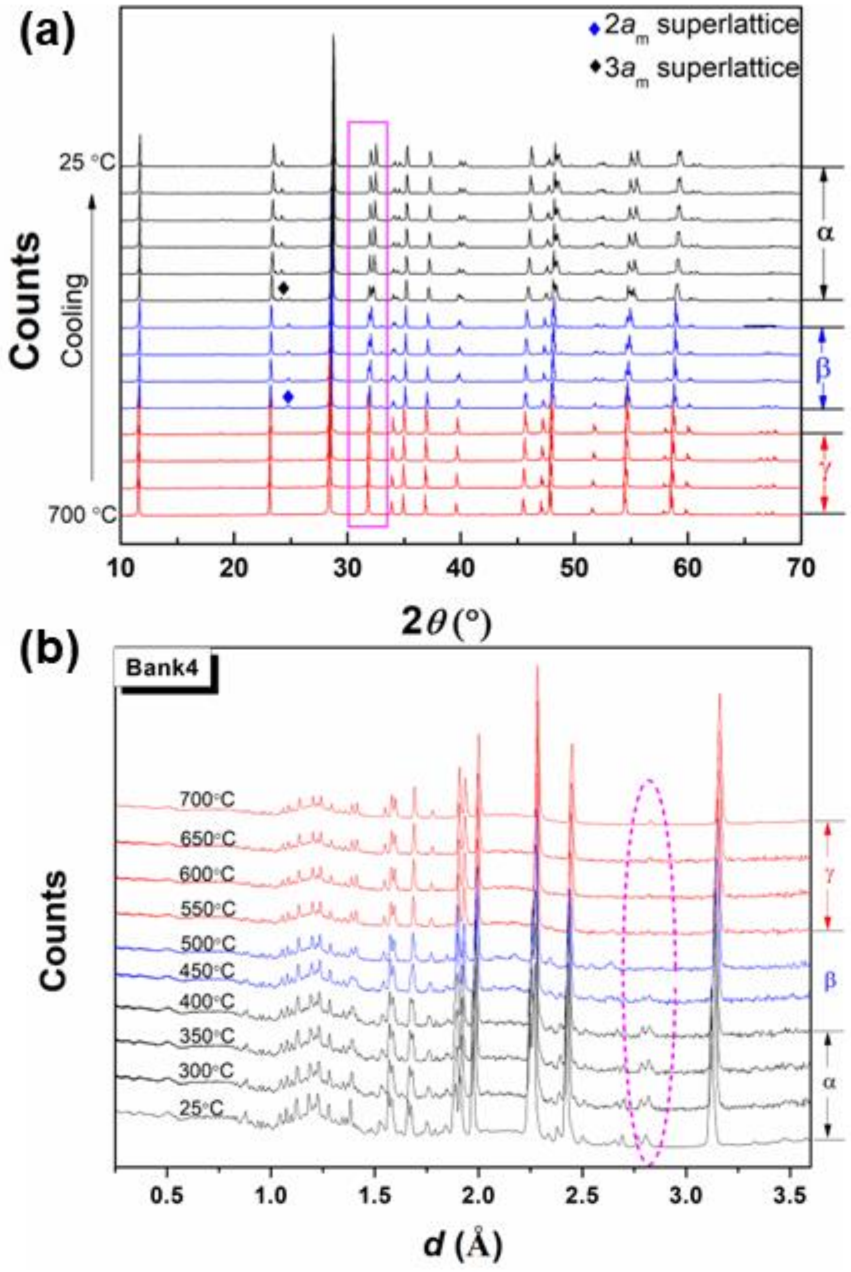

Fig. S2. Thermal evolution of (a) X-ray and (b) neutron (bank 4) diffraction patterns for BIGEVOX10 upon cooling. The phase transition is indicated by the change of (200) and (020) reflections in the mean cell (marked by dashed ellipse at $d \approx 2.78$ Å).

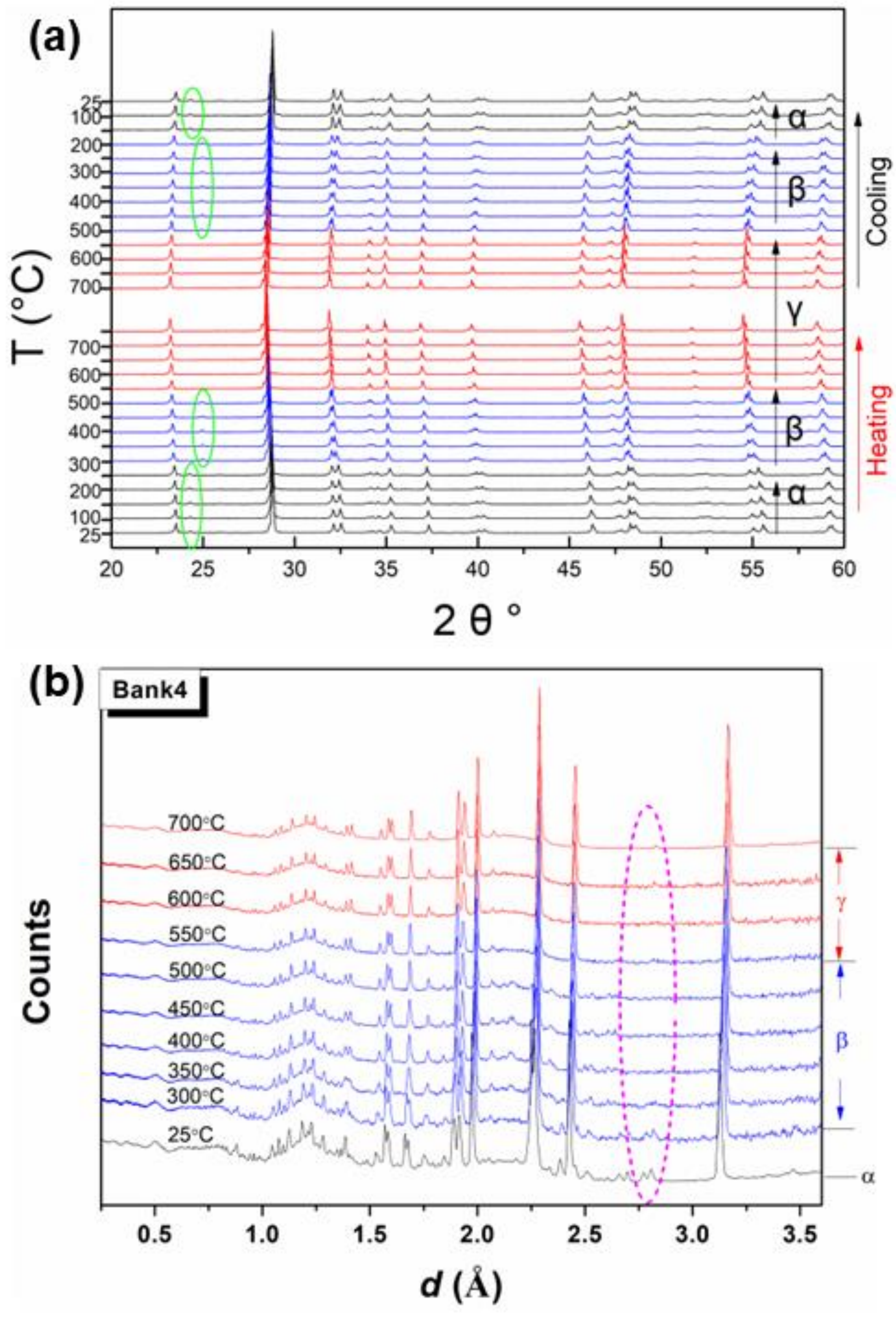

Fig. S3. Thermal evolution of (a) X-ray and (b) neutron (bank 4) diffraction patterns for BISNVOX05. The phase transition is indicated by the change of (200) and (020) reflections in the mean cell (marked by dashed ellipse at $d \approx 2.7$ Å).

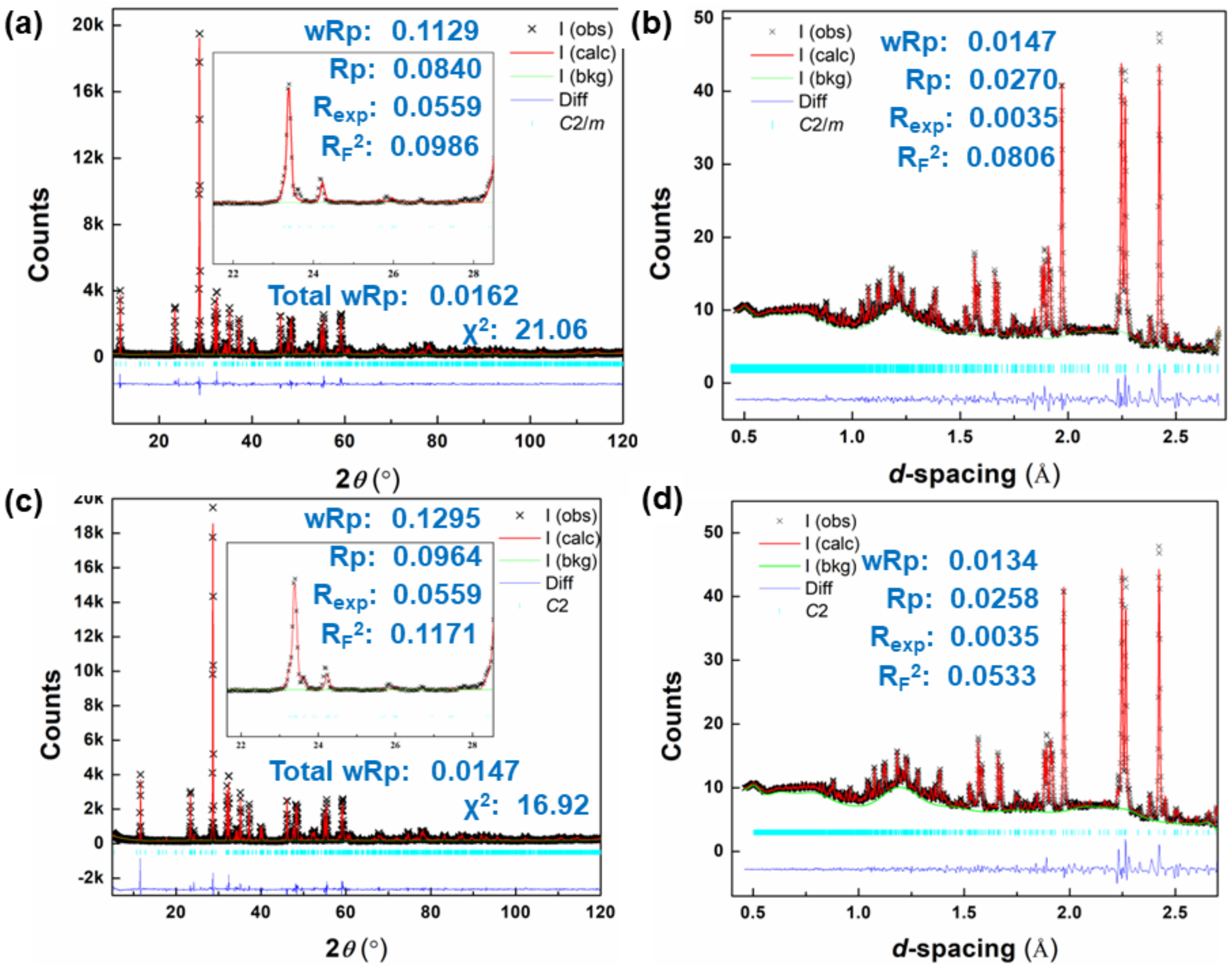

Fig. S4 Fitted diffraction profiles showing fits to (a, c) X-ray and (b, d) neutron data for BISNVOX05 at 25 °C using (a, b) *C*2/*m* and (c, d) *C*2 models.

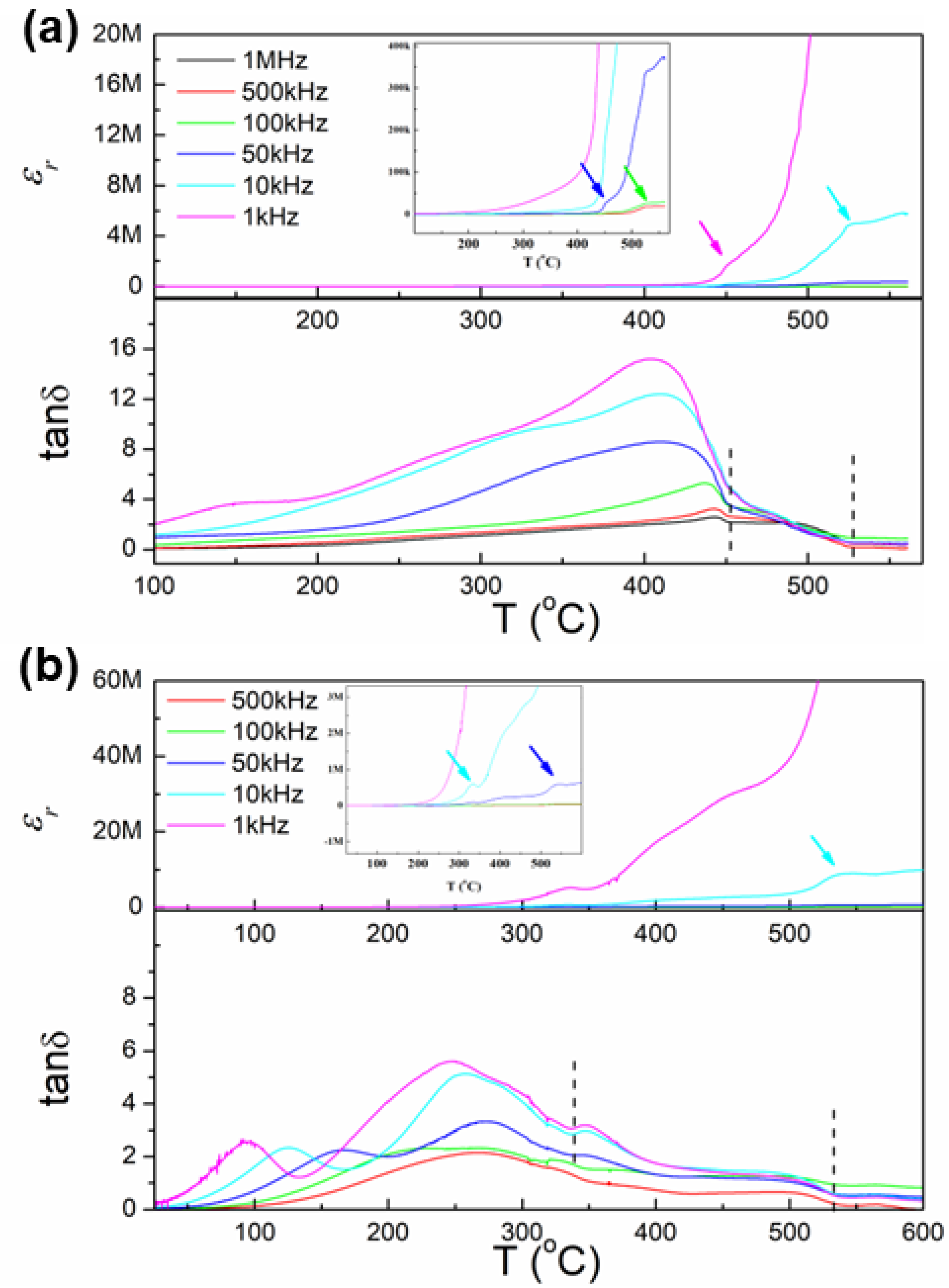

Fig. S5. High-temperature permittivity for pelletized (a) BIGEVOX10 and (b) BISNVOX05 over the frequency range from 1 kHz to 500 kHz.

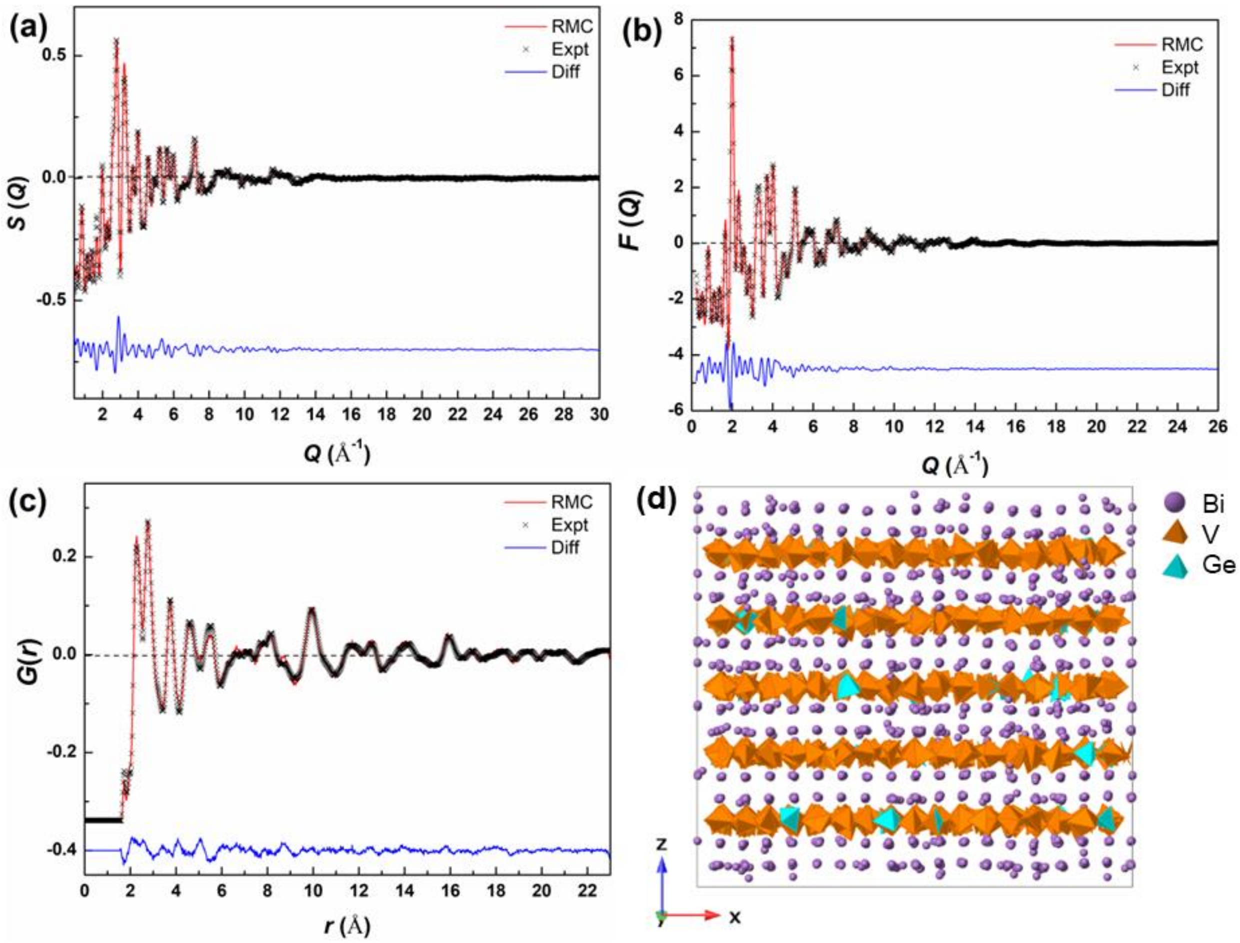

Fig. S6. Representative fitted profiles for BIGEVOX10 at 25 °C showing fits to (a) neutron $S(Q)$, (b) X-ray $F(Q)$ and (c) $G(r)$; and (d) a representative final configuration.

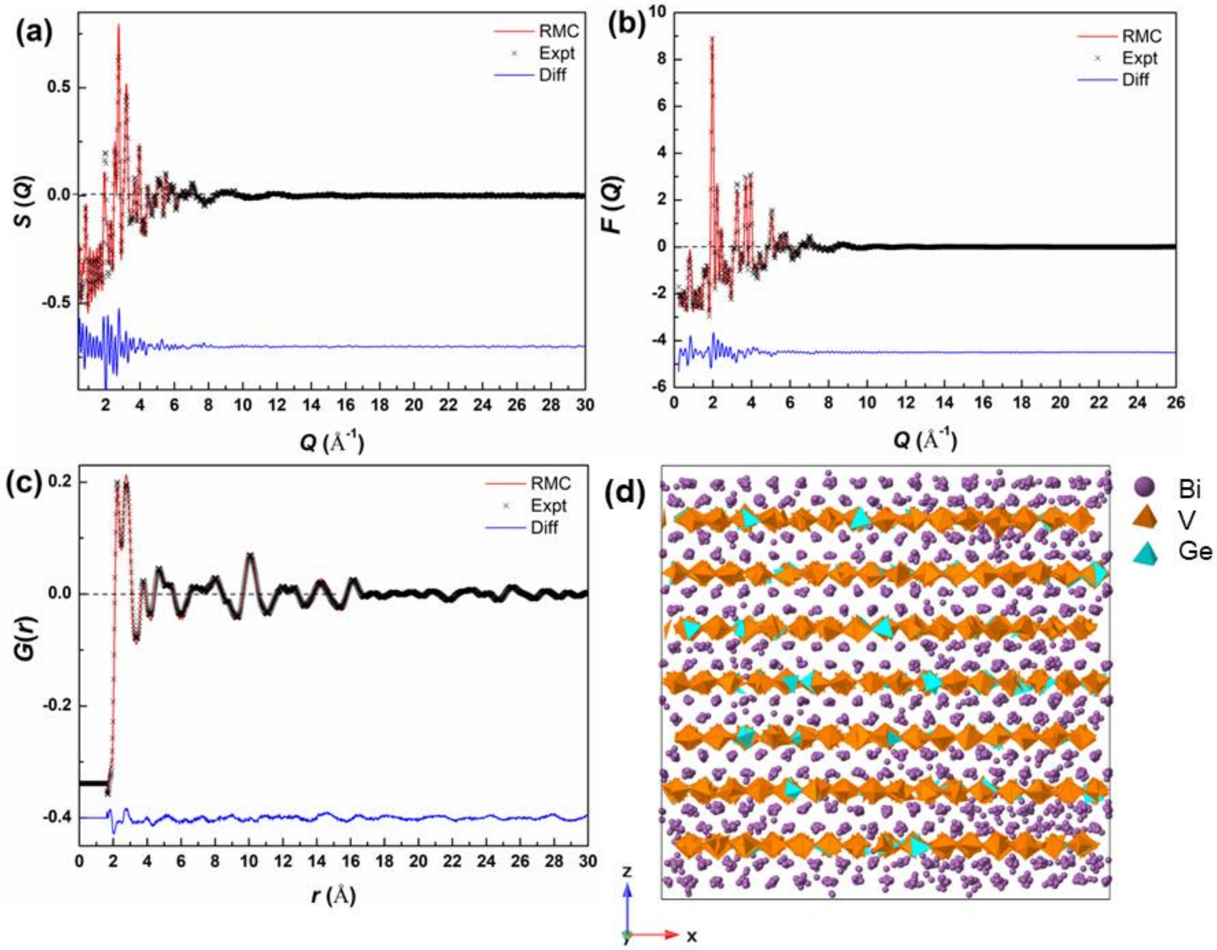

Fig. S7. Representative fitted profiles for BIGEVOX10 at 700 °C showing fits to (a) neutron $S(Q)$, (b) X-ray $F(Q)$ and (c) $G(r)$; and (d) a representative final configuration.

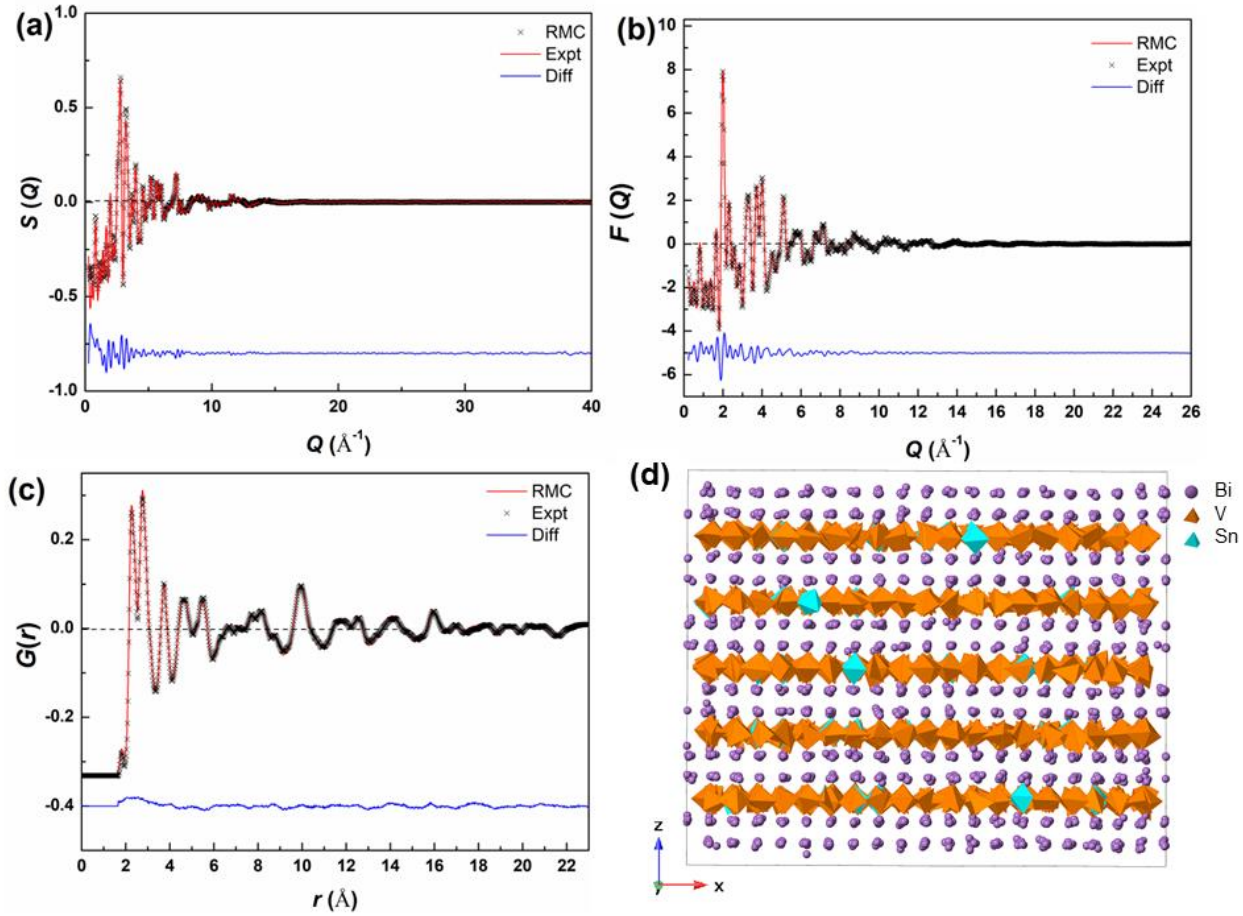

Fig. S8. Representative fitted profiles for BISNVOX05 at 25 °C showing fits to (a) neutron $S(Q)$, (b) X-ray $F(Q)$ and (c) $G(r)$; and (d) a representative final configuration.

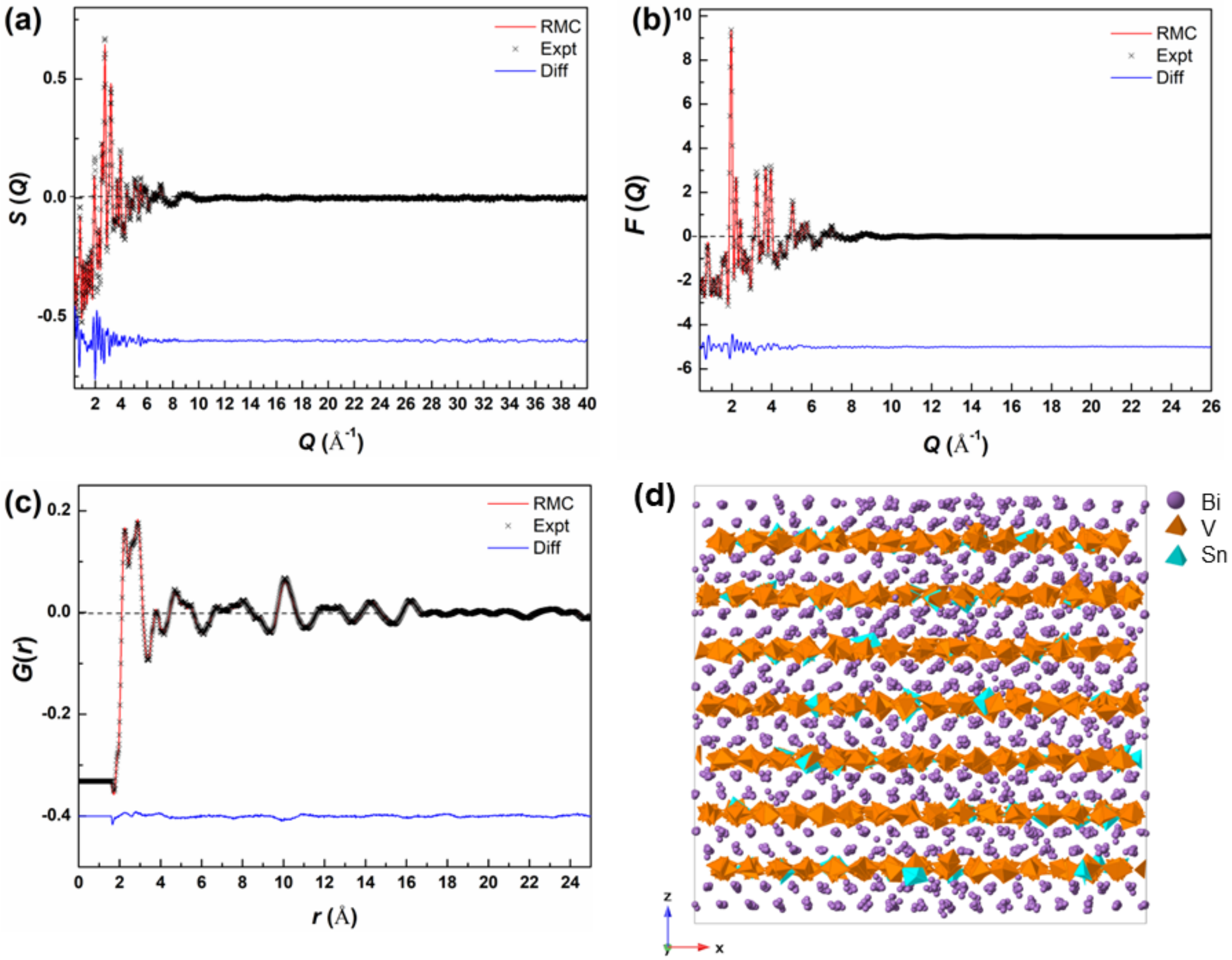

Fig. S9. Representative fitted profiles for BISNVOX05 at 700 °C showing fits to (a) neutron $S(Q)$, (b) X-ray $F(Q)$ and (c) $G(r)$; and (d) a representative final configuration.

Table S1 Comparison of theoretical and average EDX analysis values for constituent elements in $Bi_2V_{0.90}Ge_{0.10}O_{5.45}$ and $Bi_2V_{0.95}Sn_{0.05}O_{5.475}$.

Compositions	Elements	EDX analysis (%)	Theoretical (%)
BIGEVOX10	Bi	22.8 ± 2.6	23.7
	V	10.7 ± 1.6	10.7
	Ge	0.9 ± 0.8	1.2
	O	62.2 ± 6.1	64.5
BISNVOX05	Bi	24.5 ± 3.7	23.6
	V	12.6 ± 1.1	11.2
	Sn	0.5 ± 2	0.6
	O	62.5 ± 4.5	64.6

Table S2. Indices of superlattice reflections in α-BIGEVOX10

		Model			
		*Aba*2		*C*2/*m* or *C*2	
$2\theta_{obs}$ (°)	d_{obs}	*hkl*	*hklm*	*hkl*	$2\theta_{cal}$ (°)
23.53	3.78	130[a]		130	23.55
24.08	3.69		$131\bar{2}$	131	24.16
26.77	3.33	Not indexed		005	26.86

[a]Forbidden reflection

Table S3. Crystal and refinement parameters for BIGEVOX10 and BISNVOX05 at 25 °C using the $C2/m$ model.

Sample Name		BIGEVOX10	BISNVOX05
Temperature (°C)		25 °C	25 °C
Chemical formula		$Bi_2V_{0.9}Ge_{0.1}O_{5.45}$	$Bi_2V_{0.95}Sn_{0.05}O_{5.475}$
Crystal system		$C2/m$	$C2/m$
Lattice parameters (Å)		a = 5.6032(2)<br>b = 15.3213(5)<br>c = 16.5814(5)<br>β = 90.012(3)°	a = 5.6060(2)<br>b = 15.3502(5)<br>c = 16.6142(5)<br>β = 90.039(4)
Volume (Å^3)		1423.5(1)	1429.7(1)
Z		12	12
Phase fraction		95.9(1)%	100%
Density (calc) g cm^{-3}		7.814	7.803
R-factors	Neutron back scattering	R_{wp} = 0.0187<br>R_p = 0.0365<br>R_{ex} = 0.0034<br>R_F^2 = 0.1354	R_{wp} = 0.0148<br>R_p = 0.0276<br>R_{ex} = 0.0023<br>R_F^2 = 0.0815
	Neutron 90°	R_{wp} = 0.0182<br>R_p = 0.0281<br>R_{ex} = 0.0022<br>R_F^2 = 0.0944	R_{wp} = 0.0164<br>R_p = 0.0251<br>R_{ex} = 0.0035<br>R_F^2 = 0.1009
	X-ray	R_{wp} = 0.1378<br>R_p = 0.1059<br>R_{ex} = 0.0602<br>R_F^2 = 0.1804	R_{wp} = 0.1131<br>R_p = 0.0843<br>R_{ex} = 0.0559<br>R_F^2 = 0.0982
	Totals	R_{wp} = 0.0191<br>R_p = 0.0889	R_{wp} = 0.0164<br>R_p = 0.0731
No. of variables		166	157
χ^2		30.13	21.27
No. of profile points	Neut. (bs)<br>(90 °C)<br>X-ray	3790<br>2089<br>3440	3539<br>2055<br>3440

Table S4. Results of refinement significance tests between *C*2 and *C*2/*m* crystallographic models in the BIGEVOX10 and BISNVOX05 systems.

		BIGEVOX10	BISNVOX05
No. of data points		9319	9034
*C*2	No. of parameters	198	186
	R_{wp}	0.0170	0.0147
*C*2/*m*	No. of parameters	166	157
	R_{wp}	0.0191	0.0164
	R_{wp} ratio	1.12353	1.11565
	R(b, n, 0.005)	1.00186	1.00274
	Significant	Yes	Yes

Table S5. Refined atomic parameters for BIGEVOX10 at 25 °C using the *C*2 model.

Atom	Site	*x*	*y*	*z*	Occ.	U_{iso} (Å^2)
Bi1	4c	0.229(2)	0.1672(7)	0.0798(0)	1.0	0.0236(5)
Bi2	4c	0.718(2)	0.3303(9)	0.0837(6)	1.0	0.0236(5)
Bi3	4c	0.758(2)	0.1710(8)	0.2539(6)	1.0	0.0236(5)
Bi4	4c	0.263(2)	0.33173	0.2520(7)	1.0	0.0236(5)
Bi5	4c	0.238(2)	0.1741(7)	0.4143(7)	1.0	0.0236(5)
Bi6	4c	0.777(1)	0.3394(9)	0.4139(6)	1.0	0.0236(5)
M1 (V/Ge)	4c	0.33(2)	0.004(9)	0.247(7)	0.9/0.1	0.02
M2 (V/Ge)	4c	0.66(2)	-0.004(5)	0.092(7)	0.9/0.1	0.02
M2 (V/Ge)	4c	0.82(5)	-0.01(2)	0.43(1)	0.45/0.05	0.02
M2 (V/Ge)	4c	0.82(5)	-0.02(2)	0.39(1)	0.45/0.05	0.02
O(1a)	2a	0.0(0)	0.248(1)	0.0(0)	1.0	0.0043(5)
O(1b)	2b	0.5(0)	0.246(1)	0.0(0)	1.0	0.0043(5)
O(1c)	2a	0.0(0)	0.261(1)	0.5(0)	1.0	0.0043(5)
O(1d)	2b	0.5(0)	0.258(1)	0.5(0)	1.0	0.0043(5)
O(1e)	4c	-0.038(2)	0.2415(9)	0.1700(5)	1.0	0.0043(5)
O(1f)	4c	-0.014(2)	0.2455(8)	0.3197(5)	1.0	0.0043(5)
O(1g)	4c	0.518(2)	0.2519(9)	0.3344(6)	1.0	0.0043(5)
O(1h)	4c	0.497(2)	0.2358(8)	0.8359(6)	1.0	0.0043(5)
O(2a)	4c	0.173(2)	0.0922(1)	0.2403(7)	1.0	0.0167(9)
O(2b)	4c	0.803(2)	0.0921(8)	0.0811(9)	1.0	0.0167(9)
O(2c)	4c	0.788(2)	0.1100(8)	0.4171(8)	1.0	0.0167(9)
O(2d)	4c	0.657(2)	0.884(1)	0.3910(7)	1.0	0.0167(9)
O(2e)	4c	0.819(2)	-0.0896(9)	0.0681(7)	1.0	0.0167(9)
O(2f)	4c	0.158(2)	-0.087(1)	0.2029(8)	0.75(2)	0.0167(9)
O(3a)	4c	0.133(5)	-0.068(2)	0.517(1)	0.5	0.037(2)
O(3b)	4c	-0.027(5)	0.031(2)	0.343(2)	0.59(2)	0.037(2)
O(3c)	4c	0.342(2)	-0.001(1)	0.0383(6)	1.0	0.037(2)
O(3d)	4c	0.418(3)	0.027(1)	0.347(1)	1.0	0.037(2)
O(3e)	4c	0.436(5)	-0.007(2)	0.493(2)	0.5	0.037(2)
O(3f)	4c	0.432(3)	0.026(1)	0.156(1)	1.0	0.037(2)

Table S6. Refined atomic parameters for BISNVOX05 at 25 °C using the *C*2 model.

Atom	Site	*x*	*y*	*z*	Occ.	U_{iso} (Å^2)
Bi1	4c	0.217(1)	0.1573(8)	0.0798(0)	1.0	0.0163(5)
Bi2	4c	0.716(1)	0.3233(7)	0.0824(6)	1.0	0.0163(5)
Bi3	4c	0.767(2)	0.1675(9)	0.2540(6)	1.0	0.0163(5)
Bi4	4c	0.257(1)	0.3218(7)	0.2454(6)	1.0	0.0163(5)
Bi5	4c	0.233(1)	0.1649(0)	0.4111(2)	1.0	0.0163(5)
Bi6	4c	0.788(1)	0.3348(6)	0.4160(5)	1.0	0.0163(5)
M1 (V/Sn)	4c	0.26(2)	-0.01(1)	0.265(5)	0.95/0.05	0.02
M2 (V/Sn)	4c	0.67(1)	-0.005(9)	0.087(7)	0.95/0.05	0.02
M2 (V/Sn)	4c	0.89(3)	-0.01(2)	0.437(9)	0.475/0.025	0.02
M2 (V/Sn)	4c	0.66(2)	-0.01(2)	0.437(9)	0.475/0.025	0.02
O(1a)	2a	0.0(0)	0.244(1)	0.0(0)	1.0	0.0055(5)
O(1b)	2b	0.5(0)	0.244(1)	0.0(0)	1.0	0.0055(5)
O(1c)	2a	0.0(0)	0.246(1)	0.5(0)	1.0	0.0055(5)
O(1d)	2b	0.5(0)	0.248(1)	0.5(0)	1.0	0.0055(5)
O(1e)	4c	0.009(2)	0.2555(8)	0.1708(6)	1.0	0.0055(5)
O(1f)	4c	0.018(2)	0.246(1)	0.3224(6)	1.0	0.0055(5)
O(1g)	4c	0.490(2)	0.2543(9)	0.3336(7)	1.0	0.0055(5)
O(1h)	4c	0.545(1)	0.2364(9)	0.8378(6)	1.0	0.0055(5)
O(2a)	4c	0.188(2)	0.0871(9)	0.2389(7)	1.0	0.0191(9)
O(2b)	4c	0.818(2)	0.0874(9)	0.082(1)	1.0	0.0191(9)
O(2c)	4c	0.736(2)	0.1053(8)	0.4122(8)	1.0	0.0191(9)
O(2d)	4c	0.638(4)	0.878(2)	0.401(1)	0.5	0.0191(9)
O(2e)	4c	0.828(2)	-0.0935(9)	0.0707(8)	1.0	0.0191(9)
O(2f)	4c	0.177(2)	-0.1046(9)	0.2114(6)	1.0	0.0191(9)
O(3a)	4c	0.118(4)	-0.095(2)	0.538(2)	0.5	0.0348(1)
O(3b)	4c	-0.075(2)	0.031(1)	0.3392(8)	0.925	0.0348(1)
O(3c)	4c	0.353(2)	-0.003(1)	0.0412(6)	1.0	0.0348(1)
O(3d)	4c	0.439(2)	0.024(1)	0.3475(9)	1.0	0.0348(1)
O(3e)	4c	0.413(4)	0.011(2)	0.482(2)	0.5	0.0348(1)
O(3f)	4c	0.451(2)	0.019(1)	0.158(1)	1.0	0.0348(1)

Table S7. Selected contact distances for BIGEVOX10 at 25 °C in the refined *C*2 model.

Contacts	Distances(Å)	Contacts	Distances(Å)
Bi1-O(1a)	2.224(11)	M1-O(2a)	1.61(14)
Bi1-O(1b)	2.347(11)	M1-O(2f)	1.84(13)
Bi1-O(1e)	2.405(11)	M1-O(3d)	1.77(12)
Bi1-O(1h)	2.328(12)	M1-O(3f)	1.66(12)
Bi1-O(2b)	2.652(15)		
		M2-O(2b)	1.69(12)
Bi2-O(1a)	2.450(12)	M2-O(2e)	1.629(22)
Bi2-O(1b)	2.256(13)	M2-O(3c)	2.00(11)
Bi2-O(1e)	2.401(12)	M2-O(3c')	2.17(11)
Bi2-O(1h)	2.308(12)	M2-O(3f)	1.72(8)
Bi2-O(2e)	2.565(14)		
Bi2-O(2f)	2.368(18)	M3-O(2c)	1.81(28)
		M3-O(2d)	2.03(26)
Bi3-O(1e)	2.101(15)	M3-O(3a)	2.44(26)
Bi3-O(1f)	2.033(12)	M3-O(3b)	1.83(26)
Bi3-O(1g)	2.264(14)	M3-O(3e)	2.35(27)
Bi3-O(1h)	2.285(14)	M3-O(3e′)	1.87(27)
Bi3-O(2a)	2.630(15)		
		M4-O(2c)	1.98(25)
Bi4-O(1e)	2.568(14)	M4-O(2d)	1.80(26)
Bi4-O(1f)	2.327(12)	M4-O(3a)	1.72(23)
Bi4-O(1g)	2.324(15)	M4-O(3d)	2.49(27)
Bi4-O(1h)	2.471(14)	M4-O(3e)	2.40(24)
Bi4-O(2d)	2.509(15)		
Bi4-O(2f)	2.667(16)		
Bi5-O(1c)	2.365(13)		
Bi5-O(1d)	2.408(14)		
Bi5-O(1f)	2.378(14)		
Bi5-O(1g)	2.372(14)		
Bi5-O(2c)	2.709(14)		
Bi5-O(3d)	2.709(19)		
Bi6-O(1c)	2.240(13)		
Bi6-O(1d)	2.471(11)		
Bi6-O(1f)	2.423(13)		
Bi6-O(1g)	2.376(14)		
Bi6-O(2d)	2.269(13)		
Bi6-O(3a)	2.363(28)		

Table S8. Selected contact distances for BISNVOX05 at 25 °C in the refined *C*2 model.

Contacts	Distances(Å)	Contacts	Distances(Å)
Bi1-O(1a)	2.233(12)	M1-O(2a)	1.56(19)
Bi1-O(1b)	2.457(11)	M1-O(2f)	1.80(17)
Bi1-O(1e)	2.430(10)	M1-O(3b)	2.32(11)
Bi1-O(1h)	2.263(11)	M1-O(3d)	1.77(11)
Bi1-O(2b)	2.478(13)	M1-O(3f)	2.12(10)
Bi1-O(3c)	2.660(18)		
		M2-O(2b)	1.65(13)
Bi2-O(1a)	2.429(12)	M2-O(2e)	1.66(12)
Bi2-O(1b)	2.200(13)	M2-O(3c)	1.92(9)
Bi2-O(1e)	2.438(12)	M2-O(3c')	2.13(12)
Bi2-O(1h)	2.385(12)	M2-O(3f)	1.73(10)
Bi2-O(2e)	2.527(12)		
Bi2-O(2f)	2.422(12)	M3-O(2c)	2.03(24)
		M3-O(2d)	2.27(22)
Bi3-O(1e)	2.362(14)	M3-O(3a)	2.47(18)
Bi3-O(1f)	2.176(15)	M3-O(3b)	1.77(17)
Bi3-O(1g)	2.436(13)	M3-O(3e)	2.18(16)
Bi3-O(1h)	2.554(13)		
Bi3-O(2a)	2.676(15)	M4-O(2c)	1.68(24)
Bi3-O(3b)	2.681(16)	M4-O(2d)	1.88(24)
		M4-O(3a)	2.13(12)
Bi4-O(1e)	2.118(15)	M4-O(3b)	1.89(16)
Bi4-O(1f)	2.184(13)	M4-O(3d)	1.614(6)
Bi4-O(1g)	2.220(13)	M4-O(3e)	1.91(18)
Bi4-O(1h)	2.203(15)	M4-O(3e')	1.94(18)
Bi4-O(2f)	2.673(14)		
Bi5-O(1c)	2.330(11)		
Bi5-O(1d)	2.458(12)		
Bi5-O(1f)	2.279(13)		
Bi5-O(1g)	2.372(15)		
Bi5-O(3d)	2.670(17)		
Bi6-O(1c)	2.286(12)		
Bi6-O(1d)	2.517(12)		
Bi6-O(1f)	2.432(13)		
Bi6-O(1g)	2.486(13)		
Bi6-O(2d)	2.083(24)		
Bi6-O(3a)	2.480(27)		
Bi6-O(3a')	2.636(27)		

Table S9 Calculated spontaneous-polarization (P_s) values for α-phase BIGEVOX10 and BISNVOX05.

	Self-polarization (μC cm^{-2})	
Direction	BIGEVOX10	BISNVOX05
a	1.47	0.31
b	0.92	-0.61
c	-1.63	0.01

Table S10. Refined atomic parameters for BIGEVOX10 and BISNVOX05 at 700 °C.

	Atom	Site	*x*	*y*	*z*	Occ.	U_{iso} (Å^2)
BIGEVOX10	Bi	4e	0.0	0.0	0.16937(5)	1.0	0.0493(4)
	V/Ge	2b	0.5	0.5	0.0	0.9/0.1	0.107(6)
	O(1)	4d	0.0	0.5	0.25	1.0	0.0445(5)
	O(2)	4e	0.5	0.5	0.10526(28)	0.386(5)	0.0677(10)
	O(3)	32e	0.577(2)	0.066(2)	0.0291(3)	0.09063	0.111(4)
	O(4)	16n	0.5	0.292(1)	0.0915(3)	0.153(1)	0.0677(10)
BISNVOX05	Bi	4e	0.0	0.0	0. 16920(6)	1.0	0.0533(5)
	V/Sn	2b	0.5	0.5	0.0	0.95/0.05	0.134(7)
	O(1)	4d	0.0	0.5	0.25	1.0	0.0465(6)
	O(2)	4e	0.5	0.5	0.1026(3)	0.411(6)	0.075(1)
	O(3)	32e	0.584(1)	0.059(3)	0.0306(3)	0.0922	0.100(4)
	O(4)	16n	0.5	0.281(2)	0.0903(4)	0.147(2)	0.075(1)

Table S11. Selected bond lengths for BIGEVOX10 and BISNVOX05 at 700 °C.

System	Contacts	Distances(Å)	Contacts	Distances(Å)
BIGEVOX10	Bi-O(1)	2.3555(4)	M-O(2)	1.629(4)
	Bi-O(4)	2.608(4)	M-O(3)	1.819(8)
			M-O(3′)	2. 325(9)
			M-O(4)	1.643(5)
BISNVOX05	Bi-O(1)	2.3593(5)	M-O(2)	1.593(5)
	Bi-O(4)	2.600(5)	M-O(3)	1.855(10)
			M-O(3′)	2.309(11)
			M-O(4)	1.651(7)
